# Toward Understanding of the Li-Ion Migration Pathways in the Lithium Aluminum Sulfides Li_3_AlS_3_ and Li_4.3_AlS_3.3_Cl_0.7_ via ^6,7^Li Solid-State Nuclear Magnetic Resonance Spectroscopy

**DOI:** 10.1021/acs.chemmater.2c02101

**Published:** 2022-12-16

**Authors:** Benjamin B. Duff, Stuart J. Elliott, Jacinthe Gamon, Luke M. Daniels, Matthew J. Rosseinsky, Frédéric Blanc

**Affiliations:** † Department of Chemistry, University of Liverpool, Liverpool L69 7ZD, U.K.; ‡ Stephenson Institute for Renewable Energy, University of Liverpool, Liverpool L69 7ZF, U.K.; § Leverhulme Research Centre for Functional Materials Design, Materials Innovation Factory, University of Liverpool, Liverpool L7 3NY, U.K.

## Abstract

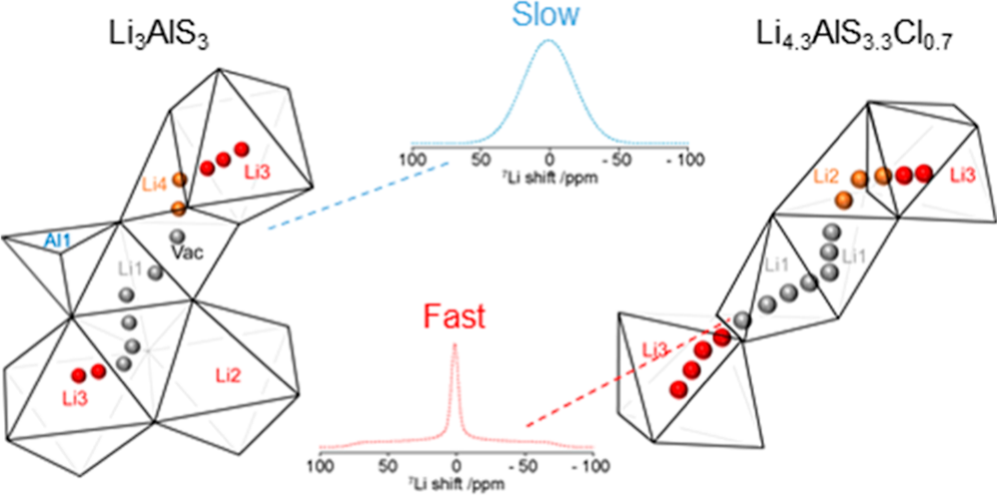

Li-containing materials providing fast ion transport pathways are fundamental in Li solid electrolytes and the future of all-solid-state batteries. Understanding these pathways, which usually benefit from structural disorder and cation/anion substitution, is paramount for further developments in next-generation Li solid electrolytes. Here, we exploit a range of variable temperature ^6^Li and ^7^Li nuclear magnetic resonance approaches to determine Li-ion mobility pathways, quantify Li-ion jump rates, and subsequently identify the limiting factors for Li-ion diffusion in Li_3_AlS_3_ and chlorine-doped analogue Li_4.3_AlS_3.3_Cl_0.7_. Static ^7^Li NMR line narrowing spectra of Li_3_AlS_3_ show the existence of both mobile and immobile Li ions, with the latter limiting long-range translational ion diffusion, while in Li_4.3_AlS_3.3_Cl_0.7_, a single type of fast-moving ion is present and responsible for the higher conductivity of this phase. ^6^Li–^6^Li exchange spectroscopy spectra of Li_3_AlS_3_ reveal that the slower moving ions hop between non-equivalent Li positions in different structural layers. The absence of the immobile ions in Li_4.3_AlS_3.3_Cl_0.7_, as revealed from ^7^Li line narrowing experiments, suggests an increased rate of ion exchange between the layers in this phase compared with Li_3_AlS_3_. Detailed analysis of spin–lattice relaxation data allows extraction of Li-ion jump rates that are significantly increased for the doped material and identify Li mobility pathways in both materials to be three-dimensional. The identification of factors limiting long-range translational Li diffusion and understanding the effects of structural modification (such as anion substitution) on Li-ion mobility provide a framework for the further development of more highly conductive Li solid electrolytes.

## Introduction

In recent years, significant progress has been made in the advancement of next-generation energy storage materials, particularly the implementation of solid-state electrolytes (SSEs) for the production of all-solid-state batteries (ASSBs).^1,2^ Current generation lithium-ion batteries with liquid electrolytes composed of a Li salt in a solvent offer high performance arising from high ionic conductivity and excellent wetting of the electrode surfaces.^3^ Adversely, liquid electrolytes contain highly volatile and flammable organic solvents which present safety issues.^4^ By comparison, the application of an SSE mitigates this safety concern and is further coupled with an increased energy density.^5^ However, overcoming the intrinsically lower ionic conductivity of solids compared with liquids as well as meeting the requirement for electrochemical stability versus electrodes remains a substantial challenge in the deployment of ASSBs.^6,7^

A room-temperature lithium conductivity target of 10^–3^ S cm^–1^ has been set for SSEs^6,8^ based on the conductivities of current generation liquid electrolytes and has now been met in several different families of materials, including garnets (Li_6.65_Ga_0.15_La_3_Zr_1.9_Sc_0.1_O_12_),^9^ glass–ceramics (Li_2_S–P_2_S_5_),^10^ thio-LISICONs (Li_9.54_Si_1.74_P_1.44_S_11.7_Cl_0.3_),^11^ halide-based SSEs (Li_3_YBr_6_),^12^ and argyrodites (Li_6.6_Si_0.6_Sb_0.4_S_5_I).^13^ Nevertheless, these materials still suffer from limitations such as one or more of the following: air and moisture sensitivity, high production costs, and poor compatibility with electrode materials. New high-performance materials can be discovered by deploying design rules developed by understanding the mechanisms of lithium ionic conduction and the limiting factors to diffusion processes in solid-state electrolytes.^14^

Higher-symmetry structures with mixed site occupancy have been shown to lead to significant improvements in ionic conductivity, the reason for which remains somewhat uncertain.^15,16^ Anion substitution in order to produce mixed anion materials has been commonly utilized in solid-state chemistry to achieve improved electrolyte stability against electrodes. In particular, halides present the advantage of being highly stable against Li metal with increased conductivities compared to pure sulfide analogues and comparatively higher oxidation potentials leading to a lower likelihood of halide oxidation.^2,11^ Moreover, as halide anions have a lower charge compared to sulfide anions, halide–sulfide substitution enables cation off-stoichiometry, which is favorable for conductivity. In particular, the Cl^–^ anion, which has a similar ionic radius to S^2–^ (167 and 170 pm, respectively),^20^ favors mixed occupancy on the anionic sites and hence disorder, leading to increased performance as shown in disordered argyrodites^17^ and the previously mentioned Li_9.54_Si_1.74_P_1.44_S_11.7_Cl_0.3_.^11^

Solid-state nuclear magnetic resonance (NMR) spectroscopy is an extremely powerful tool for the investigation of disordered materials as unlike diffraction-based methods, the technique does not depend on long-range structural order.^18^ NMR is widely used for structural determination purposes^18^ yet can also be very effectively employed for the assessment of ion dynamics and diffusion processes,^19^ complementing, for example, conductivity measurements from AC impedance spectroscopy (ACIS)^20^ or Muon spectroscopy.^21^ In particular, NMR spectroscopy offers a direct, non-destructive method to probe the mobility of Li^+^ specifically^22−26^ because of its unique inherent isotope specificity exploiting both NMR-active isotopes of lithium (^6^Li, 7.59% natural abundance, spin *I* = 1 and ^7^Li, 92.41%, *I* = 3/2) while also being suitable for powdered samples. The key to the study of dynamics by NMR lies in the wide range of timescales that can be accessed. These range from very fast motional processes in the order of 10^–12^ s^–1^ probed by measuring spin–lattice relaxation (SLR) time constants to much slower motion on the timescale of 10^–3^ s^–1^ from line shape analysis or s^–1^ in exchange spectroscopy (EXSY). NMR also allows for the extraction of site-selective dynamics information which is highly complementary to the mean structure with average occupancies of particular sites accessible by diffraction-based methods.

Static ^7^Li variable temperature (VT) NMR has been widely used to probe lithium-ion dynamics in ionic conductors, allowing for extraction of activation energies (*E*_a_) and correlation rates (τ_c_^–1^) of the Li-ion jump processes (τ_c_^–1^ is essentially the jump rate τ^–1^).^22^^7^Li VT NMR spectra can also provide qualitative insights into ion mobility in solids by identifying the sites contributing to long-range ion mobility and the pathways involved. For instance, a number of previous works^27−29^ have shown that static VT NMR spectra can support the presence of both mobile and immobile ions on the NMR timescale, which is evidenced by the superposition of resonances with different linewidths from ions moving at different rates. VT diffusion-induced ^7^Li NMR SLR rate constants in both the laboratory (*T*_1_^–1^) and rotating (*T*_1ρ_^–1^) frames allow access to quantitative information on the Li diffusion process. Additionally, the dimensionality of Li-ion mobility within the material can be extracted from the frequency dependence of the SLR rate constants as initially postulated based on theoretical calculations^30^ and recent experimental verification for Li_12_Si_7_.^31^ Two-dimensional (2D) EXSY NMR experiments are a powerful method for investigating chemical exchange in ionic conductors and allow for both qualitative observation of which inequivalent sites are involved in the ionic exchange and quantitative extraction of site-specific jump rates.^32−35^

Recently, we discovered two lithium aluminum sulfide phases, Li_3_AlS_3_^36^ and Li_4.3_AlS_3.3_Cl_0.7_,^37^ through a computational approach involving the screening and identification of new materials in the Li–Al–S phase field. We utilized ^6^Li magic angle spinning (MAS) and ^27^Al (*I* = 5/2) multiple quantum MAS (MQMAS) NMR for structure determination. Here, we expand on this work by reporting a comprehensive understanding of the lithium-ion dynamics of these phases from ^7^Li line narrowing, relaxation, EXSY data, and site-specific ^6^Li exchange. The results identify the limiting factors for Li-ion mobility in Li_3_AlS_3_ and rationalize the increased ion mobility observed in the Li_4.3_AlS_3.3_Cl_0.7_ disordered mixed anion structure.

## Experimental Section

Li_3_AlS_3_^36^ and Li_4.3_AlS_3.3_Cl_0.7_^37^ at natural abundance were synthesized according to reported solid-state synthesis procedures. ^6^Li-enriched Li_3_AlS_3_ and Li_4.3_AlS_3.3_Cl_0.7_ were prepared using the same procedure with ^6^Li-enriched Li_2_S (prepared from 95% ^6^Li-enriched Li_2_CO_3_,^16^ CortecNet, 99.7% purity) as the lithium precursor. Routine analysis of phase purity and lattice parameters was performed on a Bruker D8 Advance diffractometer with a monochromated Cu source (Kα1, λ = 1.54060 Å) in powder transmission Debye Scherrer geometry (capillary) with sample rotation. Powder X-ray diffraction patterns of the ^6^Li-enriched samples are shown in Figure S1 in the Supporting Information (SI) and are in agreement with the literature.^36,37^

Static ^7^Li VT NMR experiments were recorded on a 9.4 T Bruker Avance III HD spectrometer equipped with a 4 mm HX high-temperature MAS probe with the X channel tuned to ^7^Li at ω_0_/2π(^7^Li) = 156 MHz. All ^7^Li one-pulse NMR spectra were obtained with a hard 90° pulse at a radiofrequency (rf) field amplitude of ω_1_/2π(^7^Li) = 83 kHz. ^7^Li quantum-filtered NMR experiments were performed using the multiple-quantum filter pulse sequence π/2−τ_1_–π–τ_1_–θ–τ_2_–θ–acq with suitable phase cycling depending on whether double (θ = 54.7°) or triple (θ = 90°) quantum coherence was targeted for filtration.^38−40^ Delays τ_1_ and τ_2_ were optimized for maximum signal intensity. Hahn-echo experiments were performed using the sequence π/2−τ–π–τ–acq with hard pulses at rf field amplitudes of ω_1_/2π(^7^Li) = 83 kHz, with τ delays varied from 9 to 90 μs. All NMR spectra were obtained under quantitative recycle delays of more than 5 times the *T*_1_ time constants at each temperature. *T*_1_ time constants were measured using the saturation recovery pulse sequence (π/2)_×100_–τ–π/2–acq with increasing recovery delay values τ. For Li_3_AlS_3_, data were fitted with a bi-exponential recovery of the form 1 – *a*·exp[−(τ/*T*_1,slow_)] + *b*·exp[−(τ/*T*_1,fast_)] where *T*_1,slow_ and *T*_1,fast_ are the time constants and *a* and *b* are the proportional contributions associated with the slow and fast components of the buildup curves, respectively. For Li_4.3_AlS_3.3_Cl_0.7_, data were fitted with a stretch exponential function of the form 1 – exp[−(τ/*T*_1_)^α^] (with α ranging from 0.9 to 1). *T*_1ρ_ time constants were recorded using a standard spin-lock pulse sequence π/2–spin lock–acq (where the duration of the spin-lock pulse is incremented) at frequencies of ω_1_/2π(^7^Li) = 10, 30, and 80 kHz, and the data were fitted to a stretch exponential function of the form exp[−(τ/*T*_1ρ_)^β^] (with β ranging from 0.5 to 1). The stretch exponential was used in order to account for a distribution of τ_c_ values, temperature gradients across the sample (see below), and the inherent multi-exponential behavior for relaxation of *I* = 3/2 nuclei.^41−43^ Static ^7^Li NMR line shapes obtained at various temperatures were simulated with the solid line shape analysis tool “Sola” in Topspin to determine the ratio of the two components contributing to the line shapes as well as the quadrupolar coupling constant *C*_Q_ and the asymmetry parameter η_Q_ values. All samples for static experiments were flame-sealed in Pyrex inserts under an Ar atmosphere.

^6^Li MAS NMR experiments were performed on a 9.4 T Bruker DSX spectrometer using a 4 mm HXY MAS probe (in the double-resonance mode) with the X channel tuned to ^6^Li at ω_0_/2π(^6^Li) = 59 MHz. A 90° pulse of duration 3 μs at an rf amplitude of ω_1_/2π(^6^Li) = 83 kHz was used. The MAS frequency ω_r_/2π was set to 10 kHz. ^6^Li MAS NMR spectra were acquired under quantitative recycle delays of more than 5 times the ^6^Li *T*_1_, measured via the saturation recovery pulse sequence. Static ^6^Li *T*_1_ time constants were also recorded using the same pulse sequence. Additionally, ^6^Li MAS NMR spectra were also collected on a 20 T Bruker NEO spectrometer using a 3.2 mm HXY probe in double resonance mode, with the X channel tuned to ^6^Li at ω_0_/2π(^6^Li) = 126 MHz and at ω_r_/2π = 20 kHz with a 90° pulse duration 4.5 μs at an rf amplitude of ω_1_/2π(^6^Li) = 56 kHz for Li_3_AlS_3_ and a 90° pulse duration of 3 μs at an rf amplitude of ω_1_/2π(^6^Li) = 83 kHz for Li_4.3_AlS_3.3_Cl_0.7_.

^6^Li–^6^Li EXSY NMR experiments were performed on an 18.8 T Bruker NEO spectrometer equipped with a 1.3 mm HX MAS probe with the X channel tuned to ^6^Li at ω_0_/2π(^6^Li) = 118 MHz and a 90° pulse of duration of 3 μs at an rf amplitude of ω_1_/2π(^6^Li) = 83 kHz and under ω_r_/2π = 15 and 45 kHz. Diagonal and cross peak intensities *I*_d_ and *I*_c_, respectively, were extracted using TopSpin. The sample temperature increase due to frictional heating at faster MAS frequencies was calibrated via ^79^Br *T*_1_ measurements on the chemical shift thermometer KBr.^44^

Temperature calibrations of the 9.4 T NMR probes were performed with the chemical shift thermometers Pb(NO_3_)_2_ using ^207^Pb NMR^45,46^ and CuI and CuBr using ^63^Cu NMR.^47,48^ The errors associated with this method were calculated using the line broadening of the isotropic peak and are in the 5–20 K range. The ^6^Li and ^7^Li shifts were referenced to 10 M LiCl in D_2_O.

## Results and Discussion

### Structural Descriptions

**Figure 1:**
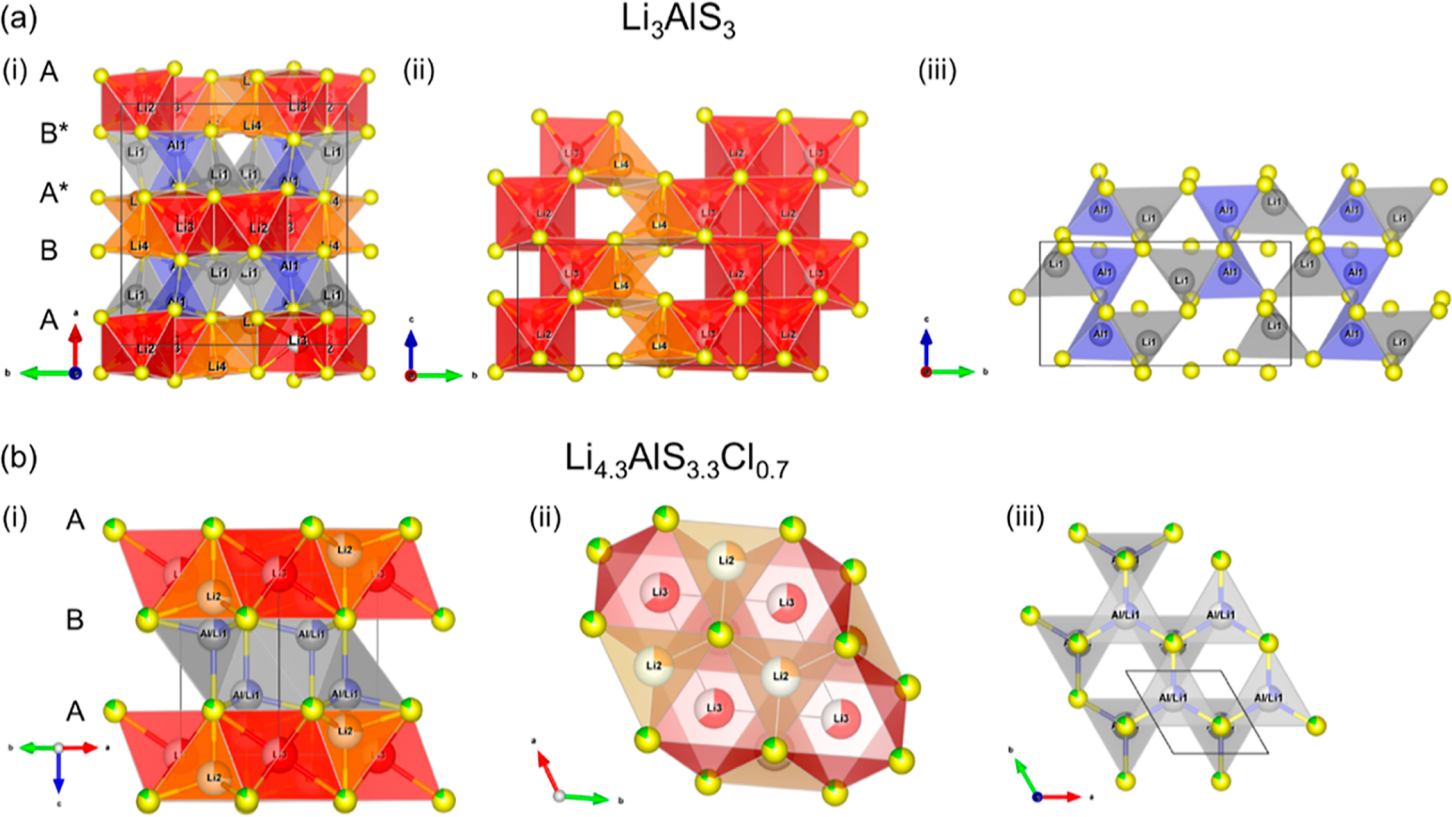
Crystal structures of (a) Li_3_AlS_3_ and (b) Li_4.3_AlS_3.3_Cl_0.7_ showing the similar hcp arrangement of sulfur atoms and the alternating mixed cation polyhedral layer and Li-only layer stacked perpendicular to the (*bc*) and (*ab*) planes in Li_3_AlS_3_ and Li_4.3_AlS_3.3_Cl_0.7_, respectively. (i) Layered view, (ii) Li only polyhedral layer, and (iii) mixed cation tetrahedral layer in the (*bc*) and (*ab*) planes for Li_3_AlS_3_ and Li_4.3_AlS_3.3_Cl_0.7_, respectively. In Li_3_AlS_3_, the A B A* B* stacking of anion layers leads to a four-layer repeat (A* and B* being the equivalent of A and B through the C centering translation). In Li_4.3_AlS_3.3_Cl_0.7_, only a two-layer repeat occurs with an A B stacking of the anion layers. Blue, orange, and red polyhedra correspond to Al tetrahedra, Li tetrahedra, and Li octahedra, respectively. Gray polyhedra correspond to Li tetrahedra from the mixed cation tetrahedral layer in Li_3_AlS_3_ and to mixed Al/Li occupied tetrahedra in Li_4.3_AlS_3.3_Cl_0.7_.

The structure of Li_3_AlS_3_^36^ consists of an hcp arrangement of sulfur atoms with an A B A* B* stacking of anion layers, giving rise to the four layer repeat (Figure 1ai) where B is the equivalent of A through the *c* glide plane and 2-fold axis symmetry operations. A* and B* are the equivalent of A and B through the C centering translation. The two different polyhedral layers are stacked alternately perpendicular to the *bc* plane (Figure 1a). In the tetrahedral layers (between A and B and between A* and B*), Li1 and Al atoms occupy 2/3 of the tetrahedral interstices between a pair of sulfur atom layers to form edge sharing tetrahedra. Between the second pairs of sulfur layers (between B and A* and between B* and A), Li2 and Li3 occupy octahedral interstices, whereas Li4 occupies a tetrahedral interstice, generating a mixed polyhedral (octahedral–tetrahedral) layer (Li-only layer). In the tetrahedral layer, Al, Li1, and vacancies are ordered in a 1:1:1 arrangement, and 2/3 of the octahedral interstices are occupied in the Li-only layer, resulting in edge sharing Li2 and Li3 octahedra, so that this structure presents a high proportion of ordered vacancies in both the tetrahedral and Li-only layers.

The substitution of Cl onto the hcp sulfur sites in Li_3_AlS_3_ gives the new Li_4.3_AlS_3.3_Cl_0.7_^37^ phase (Figure 1b), which retains the hcp arrangement of Li_3_AlS_3_, as well as the alternating tetrahedral and Li-only layer, while leading to a strong cation site disorder within the polyhedral interstices, so that Li_3_AlS_3_ is a superstructure of Li_4.3_AlS_3.3_Cl_0.7_. In the latter, the anion stacking motif is A B with only a two-layer repeat. Within the tetrahedral layer, lithium (Li1, site occupancy factor: sof_Li1_ = 0.50(1)) and aluminum (sof_Al_ = 0.25) are disordered among all the tetrahedral interstices. Within the Li-only layer, lithium and vacancies are disordered among all the octahedral (Li3, sof_Li3_ = 0.644(2)) and tetrahedral (Li2, sof_Li2_ = 0.260(2)) sites. Li1/Al and Li2 tetrahedra of the adjacent layer share a common base and form a polyhedral unit which can only host one cation. Indeed, the small hypothetical interatomic distances (*d*_Al–Li2_ = 1.543(13) Å and *d*_Li1–Li2_ = 1.274(14) Å) render the occupation of both the Li1/Al and Li2 sites of the same unit very unlikely. A summary of the various Li interatomic distances is available in Table S1.

### Static ^7^Li VT Line Narrowing NMR

**Figure 2:**
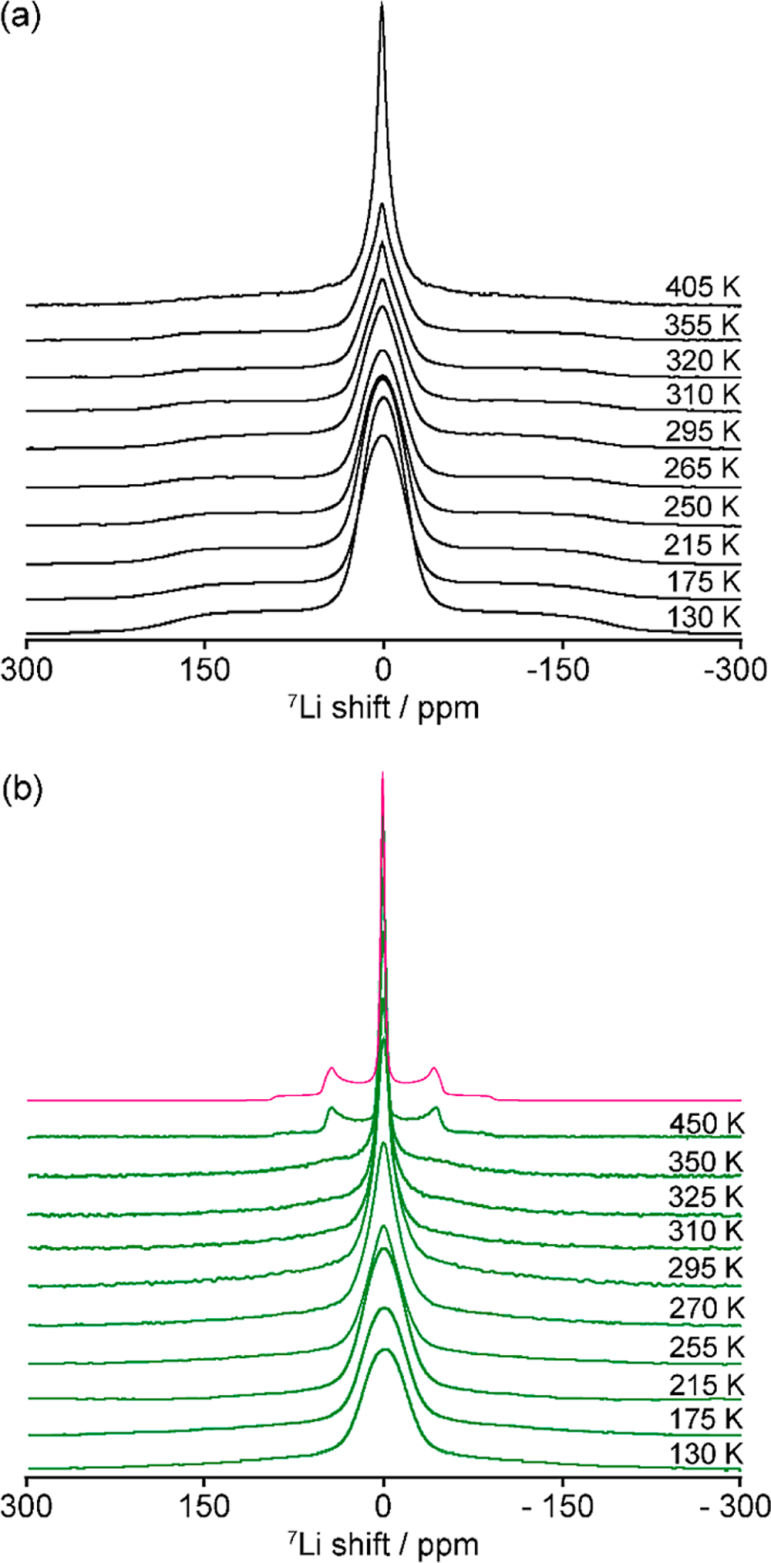
Static ^7^Li one-pulse NMR spectra of (a) Li_3_AlS_3_ and (b) Li_4.3_AlS_3.3_Cl_0.7_ as a function of temperature. The pink line displays a simulation of the static ^7^Li NMR spectrum of Li_4.3_AlS_3.3_Cl_0.7_ at 450 K using a single set of parameters describing the quadrupolar powder pattern for a spin 3/2 nucleus with a shift δ of 0 ppm, a quadrupolar coupling constant *C*_Q_ of 30 kHz, and a quadrupolar asymmetry η_Q_ of 0.1.

**Figure 3:**
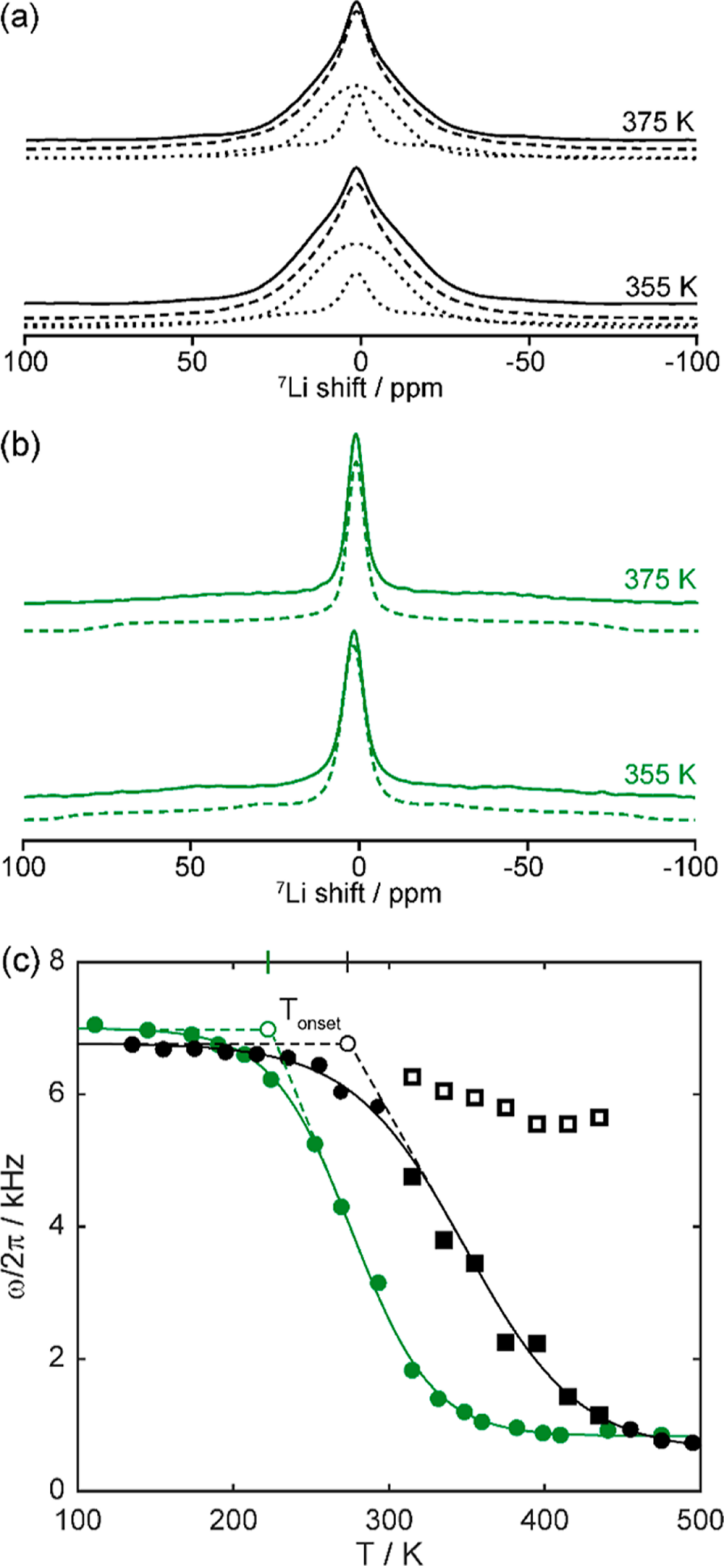
Representative static ^7^Li one-pulse NMR spectra of (a) Li_3_AlS_3_ and (b) Li_4.3_AlS_3.3_Cl_0.7_ illustrating the broad and motionally narrowed components of the NMR line shape associated with Li-ion diffusion. The experimental spectra (solid lines), total fit (dashed lines), and spectral deconvolution (dotted lines) are shown. (c) Temperature dependence of ^7^Li NMR linewidth ω(Τ)/2π of Li_3_AlS_3_ (black) and Li_4.3_AlS_3.3_Cl_0.7_ (green). In Li_3_AlS_3_, the empty and filled squares represent the broad and narrow components of the line shape observed in (a), respectively, while circles for Li_4.3_AlS_3.3_Cl_0.7_ correspond to the total line shape in (b). The onset temperatures of line narrowing *T*_onset_ are given with double-length ticks on the upper vertical axis, with dashed lines showing the tangents of the curve used to extract *T*_onset_. The solid black and green lines are fit to the data based on the sigmoidal regression given in eq 2 and are used to determine the inflection points of the respective curves.

Static ^7^Li NMR spectra of Li_3_AlS_3_ and Li_4.3_AlS_3.3_Cl_0.7_ were collected in the 130–450 K temperature range (Figure 2) to provide information on the Li-ion dynamics on the kHz timescale. At low temperatures (<250 and <200 K for Li_3_AlS_3_ and Li_4.3_AlS_3.3_Cl_0.7_, respectively), the line shape of the 1/2 ↔ −1/2 central transition strongly suggests that the linewidth is dominated by the strong ^7^Li–^7^Li homonuclear dipolar broadening of the ^7^Li spins with a static ^7^Li NMR linewidth ω/2π at a half-height of ∼6.7 kHz for Li_3_AlS_3_ and ∼7.0 kHz for Li_4.3_AlS_3.3_Cl_0.7_. This broadening is significant given that it is proportional to the square of the gyromagnetic ratio γ of the nuclear spins, which is large for ^7^Li (γ (^7^Li) = 16.5 MHz T^–1^). At these low temperatures, the materials reside in the so-called rigid lattice regime and the corresponding Li^+^-ion τ^–1^ values are smaller than ω/2π. As the sample temperature is increased, the central transition linewidths of both phases significantly decrease at the onset temperatures *T*_onset_ of motional narrowing, which occur at around 270 and 220 K for Li_3_AlS_3_ and Li_4.3_AlS_3.3_Cl_0.7_, respectively (Figure 3). This narrowing effect arises from the continuous averaging of the ^7^Li–^7^Li homonuclear dipolar coupling due to the increasing motion of the Li^+^ ions at frequencies larger than ω/2π. Importantly, the significantly lower *T*_onset_ observed for Li_4.3_AlS_3.3_Cl_0.7_ versus Li_3_AlS_3_ indicates higher Li^+^-ion mobility in the former phase, supporting the previously reported results obtained via ACIS^36,37^ where room-temperature conductivity values in the order of 10^–6^ and 10^–9^ S cm^–1^ were extracted for the respective samples.

Further sample heating above room temperature yields significantly narrower lines with ω/2π on the order of 750 Hz and a multicomponent line shape for Li_3_AlS_3_ (see below). This corresponds to τ^–1^ ≫ ω/2π in the static regime, with ^7^Li–^7^Li homonuclear dipolar coupling (fast motional regime) that is largely averaged out, and the residual linewidth is mainly governed by non-homonuclear dipolar interactions and inhomogeneities of the external magnetic field *B*_0_.^49^ The ^7^Li NMR spectrum of Li_4.3_AlS_3.3_Cl_0.7_ at 450 K displays the typical quadrupolar powder pattern of this spin *I* = 3/2 nucleus with a quadrupolar tensor in (or close to) axial symmetry, consisting of a central transition at 0 ppm and quadrupolar satellite transitions at approximately ±50 ppm corresponding to a quadrupolar coupling constant *C*_Q_ of ∼30 kHz (Figure 2b). The axial symmetry suggests that Li^+^ ions exchange between axially symmetric sites of similar orientation or between sites with different orientations averaging out two of the three components of the quadrupolar tensors. Accessing these orientations is beyond the scope of the work as this would require significant computational work^50^ capturing the complex site disorder of Li_4.3_AlS_3.3_Cl_0.7_.

Close inspection of the ^7^Li NMR spectra for Li_3_AlS_3_ between 315 and 435 K (Figure 3a for representative spectra in the middle of this temperature range) clearly reveals two contributions to the line shape. This consists of a motionally narrowed line, corresponding to highly mobile Li^+^ ions (τ^–1^ < ω/2π), superimposed on a much broader line for slower-moving ions (τ^–1^ > ω/2π). The two components display significantly different *C*_Q_ values of 58 and 15 kHz for the broad and narrow components, respectively (Figure S2). It is postulated that this two-component NMR line shape arises from ions moving along the faster diffusion pathways present in the layered structure (see below). At 315 K, approximately 12% of the Li ions present are mobile, with this percentage increasing as a function of temperature (Figure S3). This two-component line shape is not observed in the ^7^Li NMR spectra of Li_4.3_AlS_3.3_Cl_0.7_ (Figure 3b), which is ascribed to the improved mobility of the Li^+^ species facilitated by the presence of more favorable ion mobility pathways due to the introduction of disordered vacancies.

In order to confirm the presence of two superimposed quadrupolar line shapes in the ^7^Li NMR spectra of Li_3_AlS_3_, double- and triple-quantum filtration and Hahn-echo experiments were performed (Figure S4). In the double-quantum filtered spectrum, the central transition is suppressed, while the quadrupolar satellites associated with transitions between the ±3/2 <−> ±1/2 energy levels have opposite phases to one another. In Figure S4a, the presence of two sets of satellite peaks with an inverted phase can be seen. In the triple-quantum filtered spectrum, the central transition remains, while the quadrupolar satellites have an inverted phase. The triple-quantum filtered spectrum in Figure S4b displays two sets of satellite transitions with opposite phases to the central transition, with corresponding *C*_Q_ values matching well the ones obtained from the static ^7^Li one-pulse spectra obtained at VT (Figure S2). Two static ^7^Li NMR Hahn-echo experiments with different echo delays (Figure S4c) reveal efficient *T*_2_ filtering to observe a line shape dominated by a broad component at a short dephasing time which is then largely removed at a longer dephasing time where the narrower component is isolated.

Using a simple expression introduced by Waugh and Fedin^51^ correlating *T*_onset_ to *E*_a_ of the diffusion process
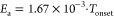
1approximate *E*_a_ values of 0.5 and 0.4 eV were estimated for Li_3_AlS_3_ and Li_4.3_AlS_3.3_Cl_0.7_, respectively, suggesting more favorable local Li^+^-ion mobility in Li_4.3_AlS_3.3_Cl_0.7_ than in the non-doped parent material as previously indicated. Moreover, the inflection points of the line narrowing curves *T*_inflection_ (Figure 3c) define the Li^+^ τ^–1^, which is of the order of (ω/2π)_rl_ (linewidth in the rigid lattice regime), and yield comparable values of ∼4.2(3) × 10^4^ s^–1^ for Li_3_AlS_3_ and ∼4.4(4) × 10^4^ s^–1^ for Li_4.3_AlS_3.3_Cl_0.7_. *T*_inflection_ values were determined from fitting the ω/2π data in Figure 3c to a Boltzmann sigmoid regression curve of the form
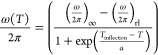
2where ω(Τ)/2π is the linewidth of the central transition at temperature *T*, (ω/2π)_∞_ is the residual linewidth at high temperature in the fast motional regime, and a is a fitting parameter that takes into a account the gradient of the slope. Importantly, the lower *T*_inflection_ value obtained for Li_4.3_AlS_3.3_Cl_0.7_ (276(6) K) than for Li_3_AlS_3_ (371(9) K) clearly indicates a faster Li^+^-ion diffusion process in the former phase.

### ^6^Li MAS NMR

**Figure 4:**
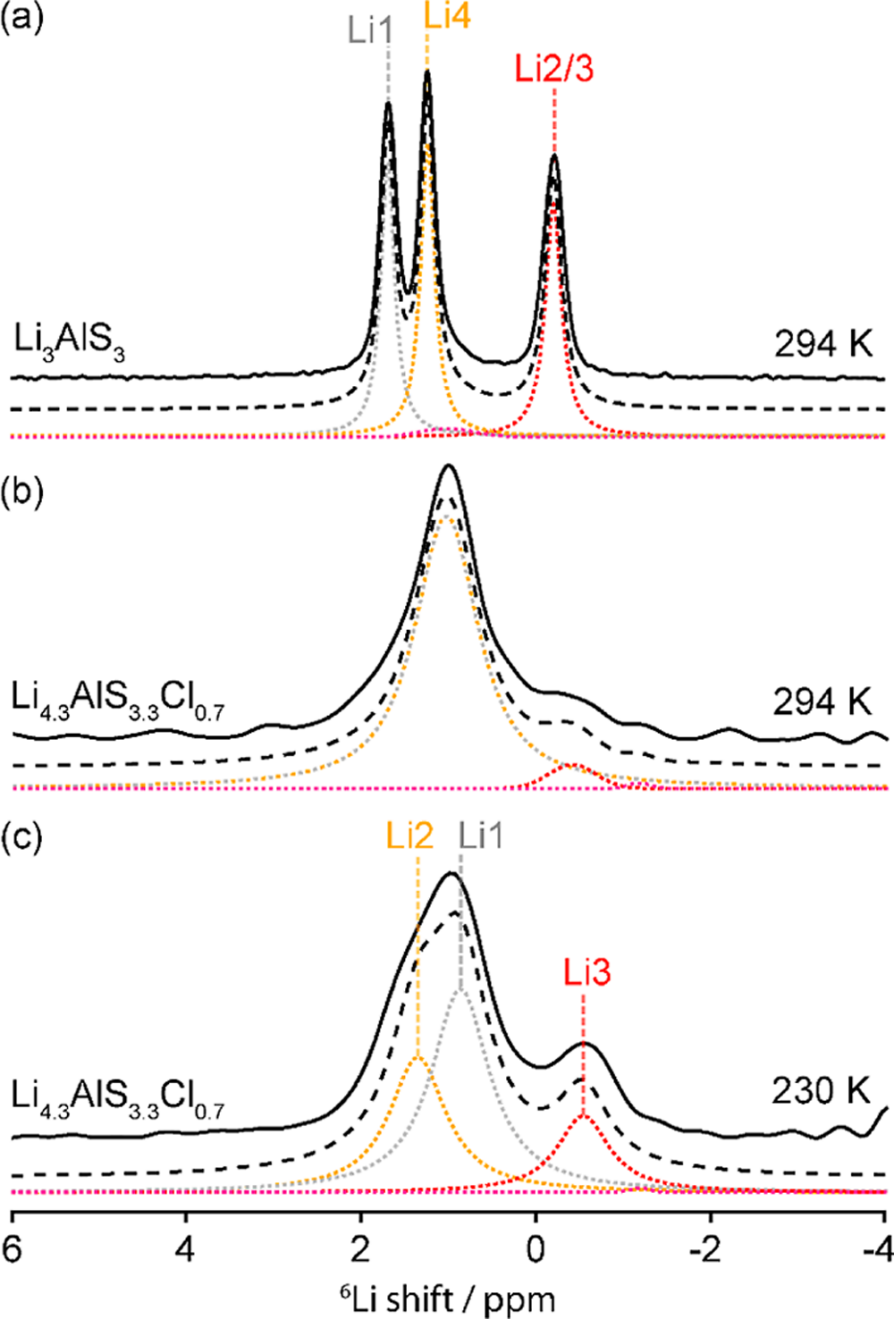
^6^Li MAS one-pulse NMR spectra collected at 9.4 T and ω_r_/2π = 10 kHz of (a) Li_3_AlS_3_ at 294 K and Li_4.3_AlS_3.3_Cl_0.7_ at (b) 294 K and (c) 230 K. The experimental spectra (full lines), total fit (dashed lines), spectral deconvolution of each Li1/Li2/Li3/Li4 signals (dotted lines), impurities Li_5_AlS_4_ in Li_3_AlS_3_, and solid LiCl in Li_4.3_AlS_3.3_Cl_0.7_ as observed in the powder X-ray diffraction patterns (pink dotted line) and spectral assignments are shown. Room-temperature data at 20 T for both phases are shown in Figure S5 and have the same resolution.

Figure 4 compares the room-temperature ^6^Li MAS NMR spectra of Li_3_AlS_3_^36^ and Li_4.3_AlS_3.3_Cl_0.7_.^37^ Li_3_AlS_3_ displays resonances at −0.2 ppm attributed to octahedrally coordinated Li2 and Li3 sites, 1.3 ppm for tetrahedral Li4, and 1.7 ppm corresponding to tetrahedral Li1,^36^ while Li_4.3_AlS_3.3_Cl_0.7_ shows an intense resonance at 1 ppm and a smaller peak at ∼−0.3 ppm assigned to the tetrahedral Li1/Li2 and octahedral Li3 sites, respectively^37^ (a smaller peak at −1.1 ppm is also visible and corresponds to residual solid LiCl).^52^ In Li_4.3_AlS_3.3_Cl_0.7_, the main signal at 1 ppm is narrow, with a peak width at a half-height of ω/2π = 50 Hz at room temperature, and suggests the presence of a motionally averaged NMR signal arising from fast Li^+^ hops between the two tetrahedral Li sites. This is not observed in the ^6^Li MAS spectrum of Li_3_AlS_3_ as Li-ion exchange between non-equivalent Li1–Li4 sites is comparatively slow on the NMR timescale. Reduced motional averaging from decreasing the Li^+^-ion mobility at 230 K revealed three different Li sites at 1.4, 0.9, and −0.55 ppm in Li_4.3_AlS_3.3_Cl_0.7_ (Figure 4c). These are assigned to tetrahedral Li2 in the Li-only layer (Figure 1b), tetrahedral Li1 in the Li/Al tetrahedral layer, and octahedral Li3 in the Li only polyhedral layer, respectively, based on the semi-empirical correlation between the lithium coordination environment and ^6^Li NMR shift.^53^ The higher resonance frequency of Li2 than Li1 arises from the large degree of bond length distortion present at Li1 (3 × 2.392(4), 2.615(6) and 3.625(6) Å), which can be considered pseudo trigonal–bipyramidal as the Li positions are strongly displaced toward one face of the tetrahedron^37^ and lead to a greater degree of chemical shielding for Li1. Note that the assignment of the tetrahedral sites in Li_4.3_AlS_3.3_Cl_0.7_ is the inverse of the spectral assignments in Li_3_AlS_3_ and this is due to the added polyhedral distortion of the Li1 position in the former, compared with the more tetrahedral Li1 in the latter. Note also that the signal intensity of the Li3 site seems to differ from the site occupancy refined against neutron powder diffraction data^37^ as, at 230 K the ^6^Li MAS NMR spectrum is in the intermediate motional regime where there is a strong interplay between signal intensity and broadening (see below).

The relative rates of site exchange occurring in Li_4.3_AlS_3.3_Cl_0.7_ can be qualitatively determined from the comparison of ^6^Li MAS spectra obtained at two different temperatures (Figure 4b,c). At 230 K, three resonances can be observed, and upon heating to room temperature, the Li1 and Li2 resonances have completely coalesced at a weighted shift average of 1 ppm. The following expression relating the frequency separation between resonances Δω/2π with the ion jump rate τ^–1^
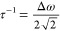
3yields a Li^+^-ion exchange rate on the timescale of τ^–1^ > 66 s^–1^ (i.e., (1.4–0.9) ppm × 59·10^6^ × π/) occurring between Li1 and Li2. Additionally, the intensity of the resonance associated with Li3 has decreased at room temperature and indicates that some exchange occurs between this octahedrally coordinated Li3 and the two tetrahedral Li1/Li2 sites at a rate of <203 s^–1^ (i.e., (1.0–(−0.55)) ppm × 59·10^6^ × π/).

**Figure 5:**
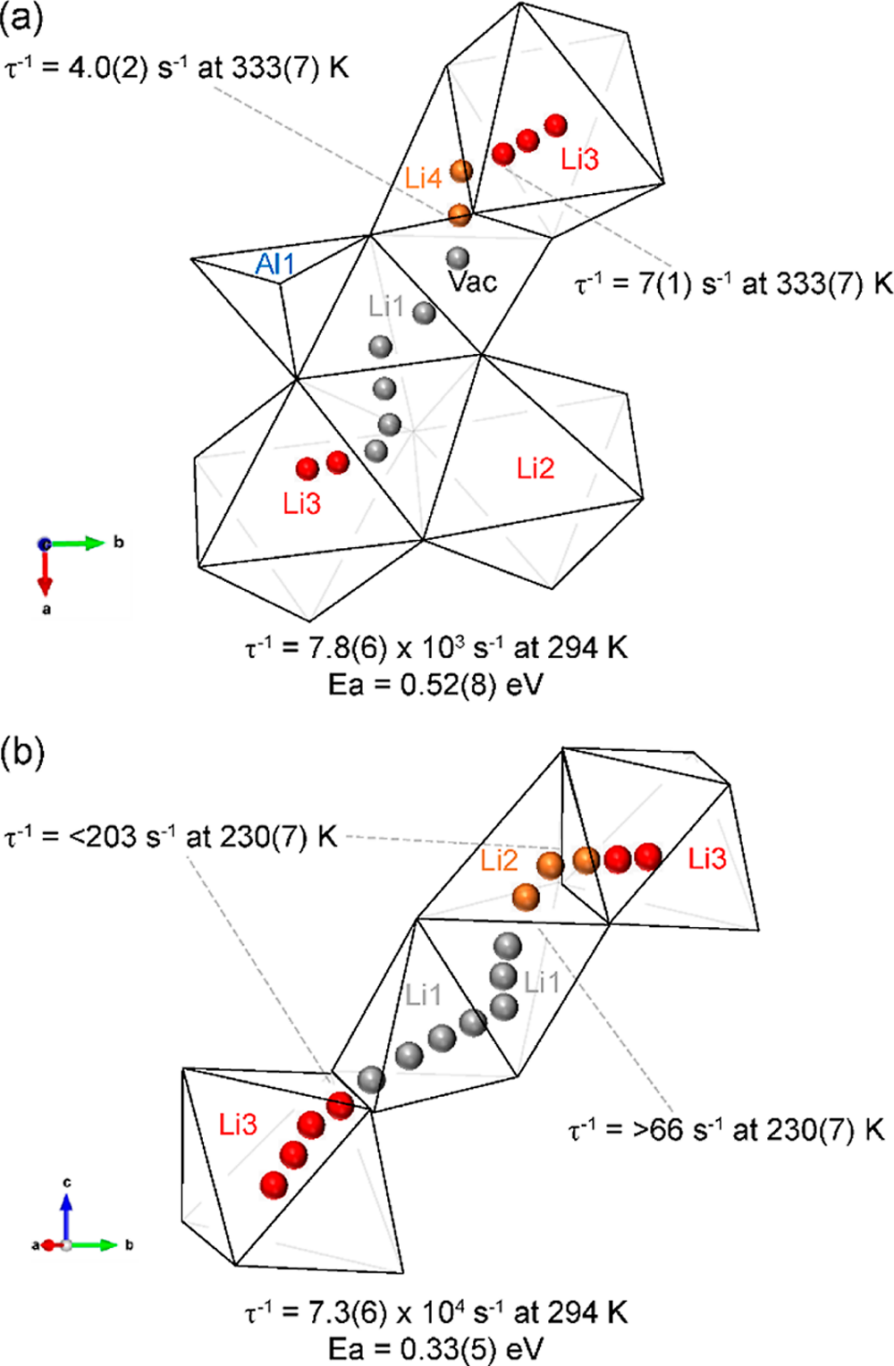
Visualization of the interlayer Li-ion migration pathway for (a) Li_3_AlS_3_ and (b) Li_4.3_AlS_3.3_Cl_0.7_ showing a schematic of the polyhedral arrangement of both phases as well as the τ^–1^ values associated with the corresponding Li-ion jumps. These values are obtained from the magnetization buildup of the 2D ^6^Li–^6^Li EXSY spectra (Figure 6) and line shape analysis of the VT one pulse ^6^Li NMR spectra (Figure 4) for Li_3_AlS_3_ and Li_4.3_AlS_3.3_Cl_0.7_. Colored spheres correspond to tetrahedral Li in the tetrahedral layer (gray), octahedral Li (red), and tetrahedral Li in the mixed polyhedral layer (orange). τ^–1^ values underneath the schematics are derived from the linear fits to the data points in Figure 8 and correspond to average jump rate values across the entire sample. Activation energies quoted are taken from the linear fit of the high-temperature data in Figure 7.

The room-temperature ^6^Li MAS spectra of the parent material reveal that the exchange between tetrahedral Li4 and octahedral Li2/3 in the mixed polyhedral layer is <197 s^–1^ (i.e., (1.3–(−0.2)) ppm × 59·10^6^ × π/), while the exchange between tetrahedral Li1 and Li4 is <52 s^–1^ (i.e., (1.7–1.3) ppm × 59·10^6^ × π/). The upper bound of this exchange rate is lower compared to the ones in the halide-substituted analogue at low temperatures and highlights increased mobility of ions exchanging between the two distinct layers in Li_4.3_AlS_3.3_Cl_0.7_. Figure 5 provides a summary of the various τ^–1^ extracted and visualizes the interlayer Li-ion migration pathway superposed on to schematics of the crystal structures.

### ^6^Li–^6^Li EXSY NMR

**Figure 6:**
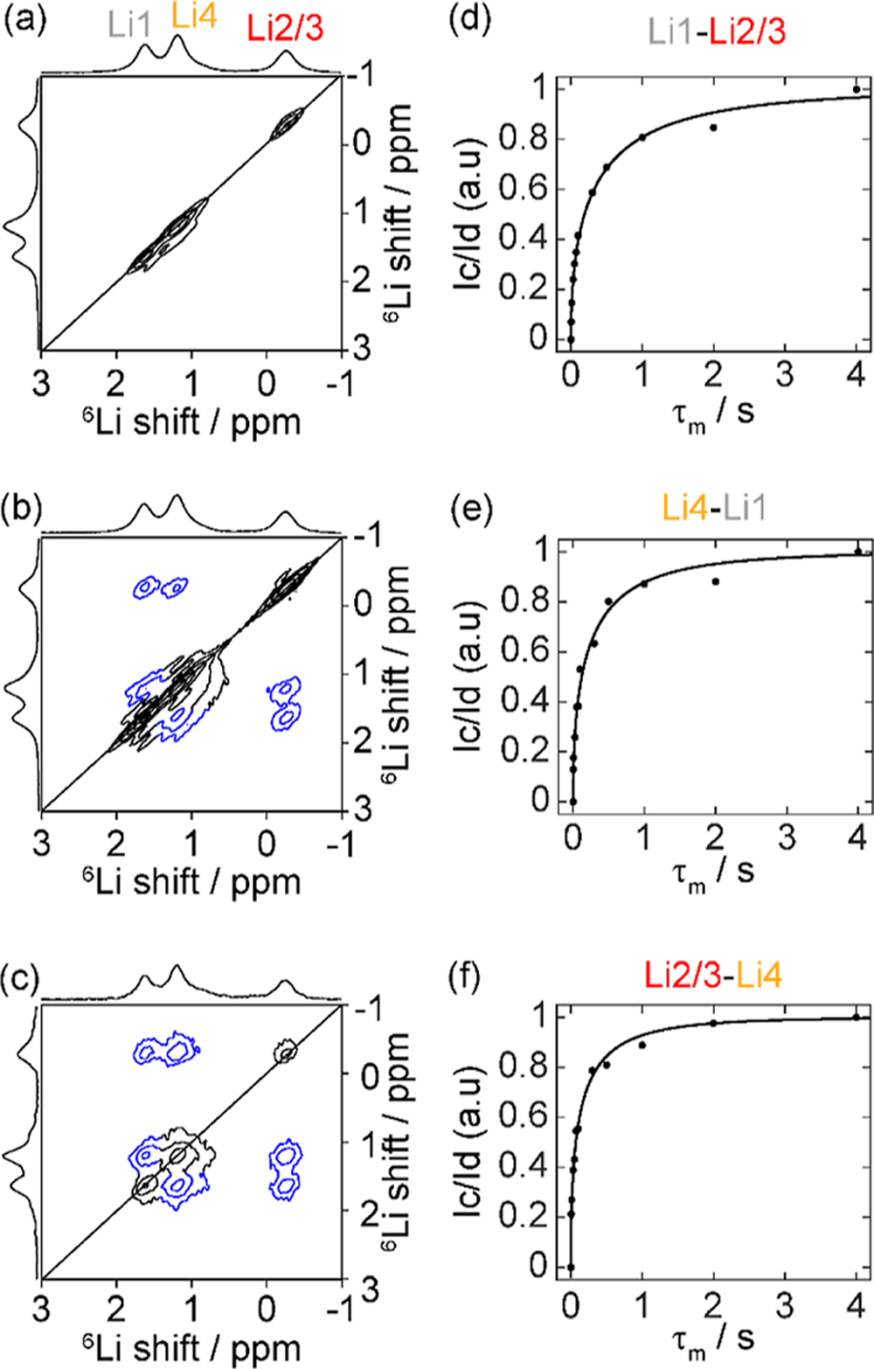
2D ^6^Li–^6^Li EXSY NMR spectra of ^6^Li-enriched Li_3_AlS_3_ recorded at ω_r_/2π = 45 kHz and mixing times τ_m_ of (a) 0, (b) 0.03, and (c) 4 s with diagonal and cross peaks shown in black and blue, respectively. The solid black lines outline the position of the diagonal. 2D ^6^Li–^6^Li EXSY NMR spectra of ^6^Li-enriched Li_4.3_AlS_3.3_Cl_0.7_ are available in Figure S8d–f plots of the cross peak buildup curves at ω_r_/2π = 45 kHz. The solid lines show the accompanying fits to eq 4, where *I*_c_ and *I*_d_ are the intensities of the observed cross and diagonal peaks, respectively.

Further insights into this pathway in Li_3_AlS_3_ were obtained from homonuclear ^6^Li–^6^Li 2D EXSY spectra on a ^6^Li-enriched sample of Li_3_AlS_3_, as a function of mixing time τ_m_ (Figure 6a–c), which exploit the high spectral resolution of the one-dimensional ^6^Li MAS spectrum of this phase. Exchange is observed experimentally in the form of off-diagonal cross peaks in the 2D EXSY spectra at the corresponding shifts and starts to emerge at around τ_m_ = 30 ms (Figure 6b) between all the sites of ^6^Li-enriched Li_3_AlS_3_. These cross peaks arise from either chemical exchange or spin diffusion from ^6^Li–^6^Li homonuclear coupling (as the sample is 95% ^6^Li-enriched) at a rate that is governed by the rate of exchange occurring during τ_m_ (Figure S6). Site-specific Li-ion correlation rates τ_c_^–1^ (and jump rates τ^–1^) can be extracted by fitting the relative intensities of diagonal (*I*_d_) and cross peaks (*I*_c_) as a function of τ_m_ to the following expression^32^
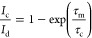
4as shown in Figure 6d–f with the extracted Li^+^ τ^–1^ values summarized in Table 1. Chemical exchange and spin diffusion processes can be differentiated by performing EXSY experiments at different MAS rates since faster MAS averages dipole–dipole interactions more efficiently and hence reduces spin diffusion. Faster cross peak buildup rates are observed for Li2/3–Li4 and Li4–Li1 when increasing the MAS rate from 15 to 45 kHz (Figures 6 and S6 and Table 1), which rules out spin diffusion in favor of supporting chemical exchange and jumps between the magnetically inequivalent sites (note that the increased rates of cross peak buildup are likely due to the temperature increase from MAS frictional heating). However, the opposite trend is observed for the Li1–Li2/3 cross peaks (i.e., a slower rate at a faster MAS frequency) and is evidence for spin diffusion between these two sites. This is unsurprising as in order for Li ions to exchange between these two sites, a three-step jump process is required along the Li1–tetrahedral vacancy–Li4–Li2/3 pathway, which would be less favored than dipolar coupling-driven spin diffusion between Li1 and Li2/3 (Figure 5).

**Table 1:** Site-Specific Li-Ion Jump Rates τ^–1^ for Li_3_AlS_3_ Extracted from ^6^Li–^6^Li EXSY Data at Two Different MAS Frequencies

		τ^–1^/s^–1^		
ω_r_/2π/kHz	*T*/K	Li1–Li2/3	Li2/3–Li4	Li4–Li1
15	309(5)	4.4(4)	5.2(6)	3.0(2)
45	333(7)	2.6(4)	7(1)	4.0(7)

The extracted τ^–1^ values clearly show the highest ion migration rates for inequivalent site exchange between Li2/3 and Li4 (*d*_Li2–Li4_ = 4.49(8) Å, *d*_Li3–Li4_ = 2.83(3) Å), which correspond to Li^+^ mobility between the tetrahedral and octahedral sites in the mixed polyhedral layer occurring through a shared face. Note that due to the lack of resolution between Li2 and Li3, it is not possible to quantify the ion migration between these two octahedral sites in the mixed polyhedral layer that form chains running along the *c*-axis. Exchange also exists between the tetrahedral Li4 and Li1 (*d*_Li1–Li4_ = 3.322(13) Å) in mixed polyhedral and tetrahedral layers, respectively, and occurs via a mutually shared face of a vacant tetrahedral site.

The lack of resolution observed in the room-temperature ^6^Li MAS spectrum of Li_4.3_AlS_3.3_Cl_0.7_ between Li1 and Li2 sites due to motional averaging, coupled with the low intensity of the Li3 resonance discussed above, prevents access to τ^–1^ values from the 2D EXSY spectra. Nevertheless, these data provide supporting evidence for the assignment of the one-dimensional ^6^Li MAS spectrum as cross peaks can be observed between the motionally averaged Li1/L2 site at ∼1 ppm and the octahedral Li3 site at ∼−0.3 ppm (Figure S8).

### ^7^Li Relaxometry

SLR rate constants in the laboratory frame *T*_1_^–1^ and the rotating frame *T*_1ρ_^–1^ were also obtained to provide further information on Li^+^ dynamics on the MHz and kHz frequency scales, respectively. Relaxation is dependent on the random fluctuation of local magnetic fields caused by the motion of atoms or functional groups. These microscopic fluctuating fields are captured by the time-dependent correlation function *G*(*t*) that contains quantitative information on the diffusion process. τ_c_ describes the timescale of these fluctuations, and in the Bloembergen–Purcell–Pound (BPP) theory, the correlation function decays exponentially and follows the equation
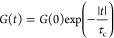
5where *G*(0) is the value of the correlation function at time *t* = 0 and is equal to the mean square of the local magnetic fields. Fourier transformation of *G*(*t*) gives the spectral density function *J*(ω_0_) which quantifies the motion at the Larmor frequency ω_0_^54,55^
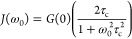
6where in this work, changes in τ_c_ are solely induced by the diffusion and motion of the spins as the increased mobility of ^7^Li nuclei with increasing temperature is the primary factor affecting the reorientation of the local magnetic fields. τ_c_^−1^ is thus temperature dependent and follows an Arrhenius relation of the type
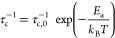
7where τ_c,0_^–1^ is the Arrhenius pre-exponential factor, *T* is the temperature, and *k*_B_ is the Boltzmann constant. The temperature dependence of the ^7^Li SLR rate constants under static conditions were collected and exploited to access the activation energy, conductivity, and dimensionality of the Li diffusion processes.

In the case of Li_3_AlS_3_, the SLR *T*_1_^–1^ buildup rates were best fitted to a bi-exponential function (Figure S9) with slow and fast relaxing components *T*_1,slow_ and *T*_1,fast_, respectively. There are two possible explanations for this behavior. First, quadrupolar nuclei with spin 3/2 such as ^7^Li will inherently relax bi-exponentially with a theoretical percentage contribution of 80 and 20% for the fast (satellite transition) and slow (central transition) relaxing contributions,^41−43^ respectively. Therefore, it is possible that the superimposed line shapes observed in the static ^7^Li VT NMR (Figure 3a) may be due to quadrupolar satellite transitions. However, if this was the case, the percentage contributions of *T*_1,slow_ and *T*_1,fast_ would be expected to remain fairly constant with temperature. In the case of Li_3_AlS_3_, the percentage contribution is 40 and 60% for *T*_1,slow_ and *T*_1,fast_, respectively, with the percentage contribution of *T*_1,slow_ increasing to 50% as the temperature increases. The second possibility is the presence of two batches of Li ions with differing τ_c_ values, giving rise to two differing values of *T*_1_. This explanation is supported by the two superimposed ^7^Li line shapes discussed previously as the double- and triple-quantum filtration and Hahn-echo experiments (Figure S4) display two sets of quadrupolar satellite transitions, arising from the two differing batches of Li ions. Therefore, we attribute the bi-exponentiality in Li_3_AlS_3_ to the presence of at least two batches of differing Li ions, which are governed by the same relaxation process but with differing values of τ_c_. In the case of Li_4.3_AlS_3.3_Cl_0.7_, the *T*_1_ buildup rates were mono-exponential as evidenced by the high stretch exponential factors α of around 0.9.

**Figure 7:**
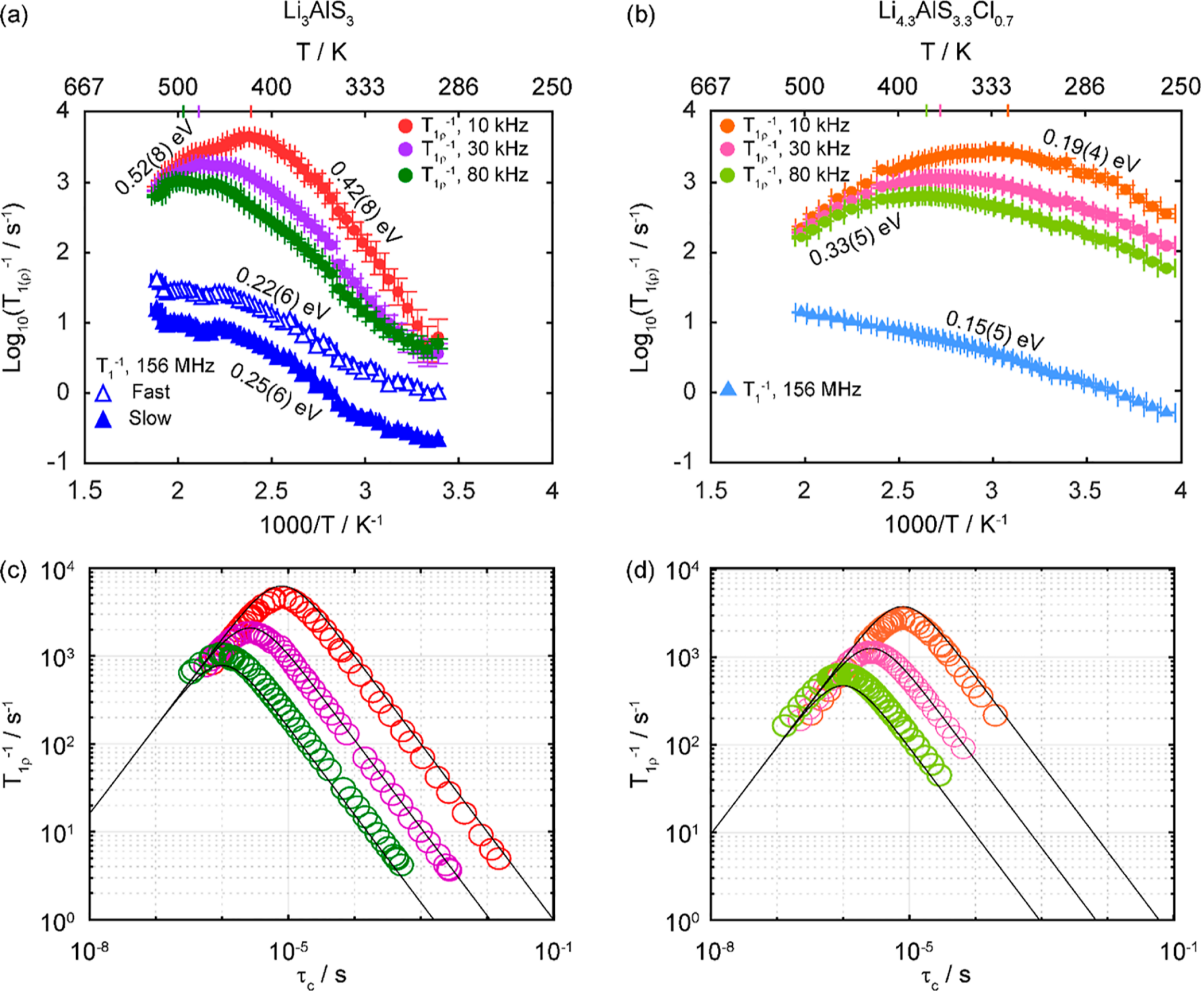
Arrhenius plots of ^7^Li NMR SLR rate constants in the laboratory frame (*T*_1_^–1^) at ω_0_/2π = 156 MHz and rotating frame (*T*_1ρ_^–1^) at ω_1_/2π = 10, 30, and 80 kHz for (a) Li_3_AlS_3_, where the filled blue triangles and empty blue triangles are the *T*_1_^–1^ SLR rate constants associated with the slow and fast components of the buildup curves, respectively, and (b) Li_4.3_AlS_3.3_Cl_0.7_. The colored ticks on the temperature scale represent the position of *T*_1ρ_^–1^ maxima. ^7^Li *T*_1ρ_^–1^ vs τ_c_ for (c) Li_3_AlS_3_ and (d) Li_4.3_AlS_3.3_Cl_0.7_. Data were collected at spin-lock frequencies of ω_1_/2π of 10 kHz (red and orange), 30 kHz (purple and pink), and 80 kHz (green and olive), and the solid lines are those obtained from eq 10 using the experimentally determined local magnetic field fluctuation terms of 1.1(6) × 10^9^ and 6.3(8) × 10^8^ Hz^2^ for Li_3_AlS_3_ and Li_4.3_AlS_3.3_Cl_0.7_, respectively. Averaged values over the three ω_1_/2π were used, resulting in the slight offsets observed at high τ_c_. ^7^Li *T*_1ρ_^–1^ vs τ_c_ data for both phases for each ω_1_/2π are given in Figure S11 for clarity.

Upon heating from room temperature to 490 K, the SLR *T*_1_^–1^ rate constants for Li_3_AlS_3_ increase from 0.22(3) to 9.4(6) s^–1^ and from 1.0(3) to 28(3) s^–1^ for the slow and fast relaxing components, respectively, while heating Li_4.3_AlS_3.3_Cl_0.7_ from 250 to 505 K results in a *T*_1_^–1^ increase from 0.46(1) to 13.6(5) s^–1^ (Figure 7). Both materials largely follow an Arrhenius behavior from which *E*_a_ barriers of 0.22(6) and 0.25(6) eV for the fast and slow components of *T*_1_^–1^ in Li_3_AlS_3_ and 0.15(5) eV for Li_4.3_AlS_3.3_Cl_0.7_ could be obtained and illustrate a significant difference between both phases. The increase in SLR *T*_1_^–1^ rate constants with higher *T* imply data in the low-temperature flank of the SLR rate constants, which are indicative of short-range motional processes, and demonstrate more energetically favorable short-range Li-ion diffusion in the more disordered Cl-doped phase.

The SLR *T*_1ρ_^–1^ data recorded at three different spin-lock frequencies ω_1_/2π are given in Figure 7. The rates initially increase with temperatures above room temperature (i.e., low-temperature flank), and activation barriers indicative of more accessible local Li^+^ jump processes for Li_4.3_AlS_3.3_Cl_0.7_ (*E*_a_ = 0.19(4) eV) than for Li_3_AlS_3_ (*E*_a_ = 0.42(8) eV) are extracted. Upon heating further, the SLR *T*_1ρ_^–1^ rate constants pass through maxima (in the 325–495 K temperature range) before decreasing (i.e., high-temperature flank) with activation barrier values for translational diffusion of 0.52(8) and 0.33(5) eV for Li_3_AlS_3_ and Li_4.3_AlS_3.3_Cl_0.7_, respectively, indicating that long-range Li^+^ diffusion is also more favorable in Li_4.3_AlS_3.3_Cl_0.7_.

SLR *T*_1ρ_^–1^ rate constants at different frequencies also provides information on the dimensionality of the Li^+^ diffusion process, and for diffusion-induced rates in solids, the high-temperature limits of the spectral density function *J*(ω_1_) have the following frequency dependence to (τ_c_/ω_1_)^0.5^, τ_c_ ln(1/ω_1_τ_c_), and τ_c_ for one-, two-, and three-dimensional diffusion processes, respectively.^30,31^ Both Li_3_AlS_3_ and Li_4.3_AlS_3.3_Cl_0.7_ materials show *T*_1ρ_^–1^ rate constants that are independent of the probe frequencies ω_1_/2π (Figure 7), which is strong evidence for the presence of three-dimensional Li^+^ mobility within both materials. This is an experimental validation of the computational prediction from ab initio molecular dynamics simulations and further evidence of the diffusion pathway revealed by the scattering density of the diffraction data,^37^ although via the direct detection of the Li^+^ ions as they proceed along the determined pathway. It is therefore postulated that Li-ion mobility occurs both between the layers and within the layers, which is in agreement with the observation of cross peaks for all Li sites in the ^6^Li–^6^Li EXSY spectra of Li_3_AlS_3_.

At the temperatures of the *T*_1ρ_^–1^ maxima, the Li^+^ τ^–1^ values are on the order of the spin-lock probe frequency ω_1_ and satisfy the following relationship^49^
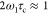
8

Li^+^ τ^–1^ values in the order of 1.3 × 10^5^–1.0 × 10^6^ s^–1^ are therefore obtained in the 420–495 and 325–380 K temperature ranges for Li_3_AlS_3_ and Li_4.3_AlS_3.3_Cl_0.7_, respectively, and the lower temperatures in the latter again demonstrate increased Li^+^ mobility.

The SLR values can be further parameterized using the following expression to extract τ_c_ from *T*_1_^–1^ rates
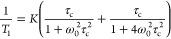
9and from *T*_1ρ_^–1^
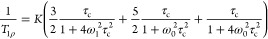
10where *K* is the local fluctuating magnetic field term in these expressions and depends on the relaxation mechanism.

For homonuclear dipolar relaxation involving spin 3/2 nuclei such as ^7^Li, *K* is proportional to the square of the dipolar coupling constant and is given by^56^
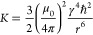
11where μ_0_ is the permeability of free space, ℏ is the reduced Planck constant, γ is the gyromagnetic ratio of the nuclear spins, and *r* is the distance between the two nuclear spins, while for quadrupolar relaxation, *K* is proportional to the quadrupolar tensor parameters and expressed as
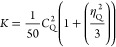
12where η_Q_ is the asymmetry parameter. Accessing τ_c_ from *T*_1_^–1^ and *T*_1ρ_^–1^ data first requires a dominant relaxation mechanism to be postulated, which for ^6,7^Li NMR is best obtained from comparing ^6^Li and ^7^Li *T*_1_ time constants under static conditions.^57^

Given the power law of 4 and quadratic dependencies of *T*_1_^–1^ rate constants on γ and the quadrupolar moment *Q* in the dipolar and quadrupolar relaxation mechanisms, respectively, a ratio of
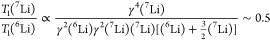
13is expected in the case of dipolar relaxation, while a ratio of
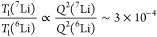
14is anticipated for a quadrupolar relaxation mechanism. Experimental *T*_1_(^7^Li)/*T*_1_(^6^Li) ratios of 0.10(3) for Li_3_AlS_3_ and 0.48(8) for Li_4.3_AlS_3.3_Cl_0.7_ were obtained at room temperature, which suggests that either cross relaxation mechanisms or a mixture of the two mechanisms contributes to the overall SLR for Li_3_AlS_3_, while dipolar relaxation mechanism dominates for Li_4.3_AlS_3.3_Cl_0.7_. The origin of this difference and of the cross relaxation may be due to a rather complex situation where the dominant relaxation mechanism changes between low and high temperatures or that the SLR rate constant measured corresponds to an average across the whole sample. Therefore, it is possible that the slow and fast moving ions observed in Figure 3 for Li_3_AlS_3_ may have these different relaxation mechanisms, as previously observed in other systems.^58,59^ Additionally, the presence of ^27^Al (a 100% abundant spin 5/2 nucleus) in close proximity with lithium ions may introduce additional heteronuclear dipolar coupling and dipolar–quadrupolar cross relaxation terms.

**Figure 8:**
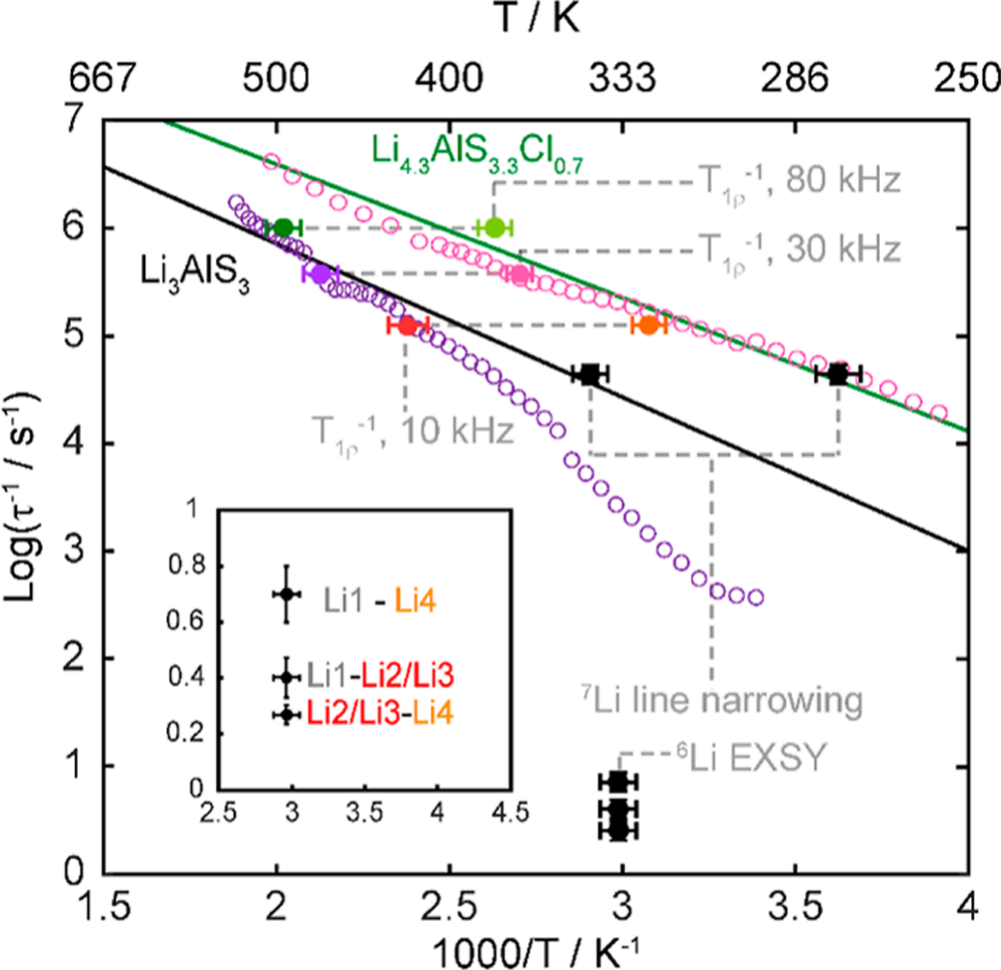
Arrhenius plot of Li-ion jump rates τ^–1^ extracted from ^7^Li linewidth, 2D ^6^Li–^6^Li EXSY experiments, and ^7^Li SLR *T*_1ρ_^–1^ rate constants. Purple and pink open circles are data points from ^7^Li SLR *T*_1ρ_^–1^ rate constants at ω_1_/2π = 30 kHz for Li_3_AlS_3_ and Li_4.3_AlS_3.3_Cl_0.7_, respectively. Red/orange (for Li_3_AlS_3_) and green/olive (for Li_4.3_AlS_3.3_Cl_0.7_) are τ^–1^ extracted from ^7^Li SLR *T*_1ρ_^–1^ maxima at ω_1_/2π = 10 and 80 kHz, respectively. The complete data sets at these frequencies have been omitted for clarity and can be found in Figures S12 and S13. Solid lines correspond to the fits to eq 7 for the experimentally determined SLR *T*_1ρ_^–1^ maxima and the value of τ^–1^ extracted from ^7^Li linewidth experiments for Li_3_AlS_3_ (black) and Li_4.3_AlS_3.3_Cl_0.7_ (green). The inset demonstrates a magnified view of the region at which τ^–1^ values extracted from 2D ^6^Li–^6^Li EXSY experiments of Li_3_AlS_3_ reside.

At the ^7^Li *T*_1ρ_^–1^ maxima, substituting eq 8 into eq 10 enables experimental determination of the local fluctuating magnetic field term *K*. A value of 1.1(6) × 10^9^ Hz^2^ (averaged over the three consistent values of *K* for the three different ω_1_/2π frequencies used) was extracted for Li_3_AlS_3_ and lies between the calculated *K* terms for the dipolar (2 × 10^7^ Hz^2^ using eq 11 and the closest Li–Li jump distance of 3.3 Å at room temperature for Li_3_AlS_3_ based on the crystal structure) and quadrupolar (4 × 10^9^ Hz^2^, eq 12) relaxation mechanisms as one would expect for cross relaxation. In Li_4.3_AlS_3.3_Cl_0.7_, the experimental (averaged) *K* value of 6.3(8) × 10^8^ Hz^2^ compares favorably with the dipolar dominated relaxation value of 5 × 10^7^ Hz^2^ (using a static 2.4 Å Li–Li jump distance for Li_4.3_AlS_3.3_Cl_0.7_). These experimentally determined *K* values are then used to convert experimental *T*_1ρ_^–1^ values to τ_c_ values at each temperature using eq 10 (Figure 7) and allows access to τ_c_ and NMR-derived jump rates τ^–1^ at all temperatures (Figure 8).

### NMR-Derived Li^+^-Ion τ^–1^ Values

NMR-derived jump rates τ^–1^ obtained from NMR line narrowing experiments (Figure 3), EXSYs (Figure 6), and relaxometry experiments (Figure 7) are plotted against reciprocal temperature in Figure 8 for ω_1_/2π = 30 kHz (data for ω_1_/2π = 10 and 80 kHz are given in Figures S12 and S13, respectively). Overall, Li-ion jump rates τ^–1^ are significantly lower for Li_3_AlS_3_ than for Li_4.3_AlS_3.3_Cl_0.7_ and reinforce the increased Li-ion mobility in the latter phase as revealed from the known ACIS impedance data.^36,37^ For both phases, there is an excellent agreement between τ^–1^ obtained from ^7^Li line narrowing spectra and relaxometry data, and fitting those to an Arrhenius equation yields activation barriers of 0.29 (0.21–0.41) and 0.29 (0.25–0.37) eV for Li_3_AlS_3_ and Li_4.3_AlS_3.3_Cl_0.7_, respectively. The values of the activation energies here are lower than the previously reported values from bulk conductivity observed by ACIS (0.48(1) and 0.33(1) eV), which is a common experimental observation^60−63^ due to the largely different approaches used where NMR spectroscopy determines the activation barrier for Li-ion mobility to neighboring sites over a much shorter length scale, whereas impedance measurements probe longer-range translational Li diffusion. Additionally, NMR also accesses unsuccessful Li-ion hops, which is likely prevalent in Li_3_AlS_3_ and Li_4.3_AlS_3.3_Cl_0.7_ as the Li^+^ sites are partially occupied. While the same activation energy is extracted from Figure 8 for both phases, the errors are particularly large due to the combination of various methods used. Hence, the energy barriers obtained from ^7^Li line narrowing and SLR experiments are likely to be more informative. A summary of the activation energies obtained via the various spectroscopic methods is available in Table S2.

The Li_3_AlS_3_ τ^–1^ values obtained from the EXSYs are notably lower than those extracted from the other approaches used. This is attributed to site-specific diffusion processes captured by the EXSY experiments compared to the information averaged across all Li sites only accessible from ^7^Li line narrowing and SLR measurements. The two components of the static ^7^Li NMR line shape observed in line narrowing experiments of Li_3_AlS_3_ can therefore be attributed to slow-moving ions (τ^–1^ ≪ ω/2π, broad component in ^7^Li static NMR spectra) associated with non-equivalent site exchange occurring between the layers (*d*_Li1–Li2_ = 4.07(13) Å, *d*_Li1–Li3_ = 3.12(3) Å, *d*_Li1–Li4_ = 3.322(13) Å) and between tetrahedral Li4 and octahedral Li2/3 sites in the mixed polyhedral layer (*d*_Li2–Li4_ = 4.49(8) Å, *d*_Li3–Li4_ = 2.83(3) Å), while the fast-moving ions (τ^–1^ ≫ ω/2π, narrow component) can be assigned to exchange occurring within the layers themselves along the *c*-axis between equivalent sites. Since the ion mobility mechanism is three-dimensional, migration between the layers is crucial for long-range translational diffusion since it connects the different diffusion channels along the *c*-axis so as to avoid any blockages that may occur in the diffusion pathways. We note that the calculated τ^–1^ values for Li_3_AlS_3_ derived from BPP theory deviate slightly from the expected trend observed from the experimentally determined τ^–1^ (through ^7^Li line narrowing and *T*_1ρ_^–1^ maxima) at low temperatures. A possible explanation for this observation is that since the Li-ion τ^–1^ for the various different sites in Li_3_AlS_3_ are expected to differ, it seems that the dependence of τ^–1^ on the temperature should also differ for the different sites. Additionally, it is possible that there is a significant number of unsuccessful Li-ion jumps given that ion mobility between non-equivalent sites is limited. The large distribution of Li-ion τ^–1^ values, originating from both fast and slow Li-ion mobility, likely results in a complex relaxation profile that deviates from the single τ^–1^ approach in the BPP model used, and the potential use of other models such as the Cole–Cole,^42^ Cole–Davidson,^43^ or a related model^64^ could potentially further rationalize this behavior. However, these models are used in order to account for a distribution of τ_c_ values across various sites and pathways, while in the case of Li_3_AlS_3_, we are probing one Li-ion pathway with two largely different τ_c_ values. Moreover, in this work, we are interested in comparing the diffusion pathways in Li_3_AlS_3_ and Li_4.3_AlS_3.3_Cl_0.7_, two closely related phases with connected chemistries, and we have chosen to exploit the BPP model as the relaxation behavior of Li_4.3_AlS_3.3_Cl_0.7_ is close to being mono-exponential.

τ^–1^ can then be used to derive conductivities σ_NMR_ from NMR data using the combined Nernst–Einstein and Einstein–Smoluchowski equations
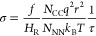
15where *f* and *H*_R_ are the correlation factor and Haven ratio, respectively (*f*/*H*_R_ is assumed to be smaller than 1 for correlated motion as per our previous ab initio calculations on Li_4.3_AlS_3.3_Cl_0.7_^37^), *N*_CC_ is the number of charge carriers per unit cell volume (based on unit cell volumes obtained from diffraction of 1014 Å^3^ for Li_3_AlS_3_^36^ and 83.5 Å^3^ for Li_4.3_AlS_3.3_Cl_0.7_^37^), *q* is the ionic charge of Li, *r* is the closest Li–Li jump distance as given above, and *N*_NN_ is the number of neighboring Li sites (6 for the three-dimensional diffusion here). Obtaining *f* and *H*_R_ is beyond the scope of this work as exemplified for the extremely well-studied Li garnet,^65^ and we have therefore chosen to provide the upper boundary of the extrapolated conductivity values. At 303 K, these are 4(3) × 10^–8^ and 3(2) × 10^–6^ S cm^–1^ for Li_3_AlS_3_ and Li_4.3_AlS_3.3_Cl_0.7_, respectively, which are in cautious agreement with the values determined by ACIS (10^–9^ and 10^–6^ S cm^–1^ for Li_3_AlS_3_^36^ and Li_4.3_AlS_3.3_Cl_0.7_,^37^ respectively).

## Conclusions

We employed a range of complementary NMR approaches focusing on both ^6^Li and ^7^Li nuclear spins to capture the Li^+^ dynamics of two newly discovered Li-containing materials, Li_3_AlS_3_ and Li_4.3_AlS_3.3_Cl_0.7_. In the parent Li_3_AlS_3_ phase, the two-component line shapes of the ^7^Li static NMR spectra demonstrate the existence of fast- and slow-moving Li^+^. The slow-moving ions were identified to diffuse between non-equivalent sites and are located between the two distinct tetrahedral and mixed polyhedral layers of the material, as captured by 2D ^6^Li–^6^Li EXSY spectra on ^6^Li-enriched Li_3_AlS_3_. In Li_4.3_AlS_3.3_Cl_0.7_, the single component observed in the static ^7^Li NMR spectra and the absence of the broad component contribution to the ^7^Li line shape indicate that the exchange of Li ions between the layers facilitates an overall increase in conductivity, as confirmed by the NMR-derived conductivities, and is associated with accelerated Li^+^ diffusion of the immobile ions of the parent Li_3_AlS_3_ material. This arises from the introduction of disordered octahedral lithium vacancies in the mixed polyhedral layer of Li_4.3_AlS_3.3_Cl_0.7_ that opens up Li-ion diffusion pathways not available by the presence of ordered vacancies in the tetrahedral layer of Li_3_AlS_3_. The frequency dependence of the SLR rate constants provides direct experimental evidence of the three-dimensional Li^+^ diffusion previously proposed. This dimensionality is, therefore, an important factor to increase ion mobility by accelerating ion exchange between layers to avoid any bottlenecks that occur via faster diffusion pathways along the *c*-axis for Li_3_AlS_3_ and the *ab* plane for Li_4.3_AlS_3.3_Cl_0.7_. Overall, this work illustrates the importance of ^6^Li and ^7^Li NMR data in accessing dynamics to understand Li-ion mobility pathways in structures that are structurally related by order/disorder.

## Supporting Information Available

Powder XRD patterns of ^6^Li-enriched Li_3_AlS_3_ and Li_4.3_AlS_3.3_Cl_0.7_, static ^7^Li VT NMR of Li_3_AlS_3_ displaying full spectral region, percentage of mobile ions in Li_3_AlS_3_ as a function of temperature, multiple-quantum and Hahn echo ^7^Li NMR spectra of Li_3_AlS_3_, ^6^Li MAS NMR of Li_3_AlS_3_ and Li_4.3_AlS_3.3_Cl_0.7_, ^6^Li–^6^Li EXSY buildups and fits of Li_3_AlS_3_, 2D EXSY of Li_4.3_AlS_3.3_Cl_0.7_, comparison of fits for *T*_1_ magnetization buildups of Li_3_AlS_3_ and Li_4.3_AlS_3.3_Cl_0.7_, ^7^Li relaxometry BPP curves, and ^7^Li jump rate plots displaying calculated values at spin lock ω_1_/2π = 10 and 80 kHz (PDF)

## Notes

Data availability: The research data supporting this publication including XRD patterns, static ^7^Li VT, ^6^Li MAS, ^6^Li–^6^Li EXSY, and ^7^Li relaxometry experiments as well as jump rate plots are accessible from the University of Liverpool Data catalogue: https://doi.org/10.17638/datacat.liverpool.ac.uk/1977.
