# Supplementary material for: Toward Understanding of the Li-Ion Migration Pathways in the Lithium Aluminum Sulfides Li3AlS3 and Li4.3AlS3.3Cl0.7 via 6,7Li Solid-State Nuclear Magnetic Resonance Spectroscopy
Source: Chem Mater. 2022 Dec 16;35(1):27–40. doi: 10.1021/acs.chemmater.2c02101 (PMC9835825; doi:10.1021/acs.chemmater.2c02101)
Supplement: Supplementary file 1 — cm2c02101_si_001.pdf [file cm2c02101_si_001.pdf]

Supporting Information For

Towards Understanding of the Li Ion Migration Pathways in the Lithium Aluminium Sulphides Li_3AlS_3 and $Li_{4.3}AlS_{3.3}Cl_{0.7}$ via $^{6,7}Li$ Solid-State Nuclear Magnetic Resonance Spectroscopy

Benjamin B. Duff,[a,b] Stuart J. Elliott,[a,#] Jacinthe Gamon,[a,$] Luke M. Daniels,[a] Matthew J. Rosseinsky,[a,c] and Frédéric Blanc[a,b,c*]

[a] Department of Chemistry, University of Liverpool, L69 7ZD Liverpool, United Kingdom

[b] Stephenson Institute for Renewable Energy, University of Liverpool, L69 7ZF Liverpool, United Kingdom

[c] Leverhulme Research Centre for Functional Materials Design, Materials Innovation Factory, University of Liverpool, L7 3NY Liverpool, United Kingdom

Present addresses:

[#] Molecular Sciences Research Hub, Imperial College London, London W12 0BZ, United Kingdom

[$] CNRS, Université Bordeaux, Bordeaux INP, ICMCB UPR 9048, Pessac 33600, France

*Corresponding Author: frederic.blanc@liverpool.ac.uk

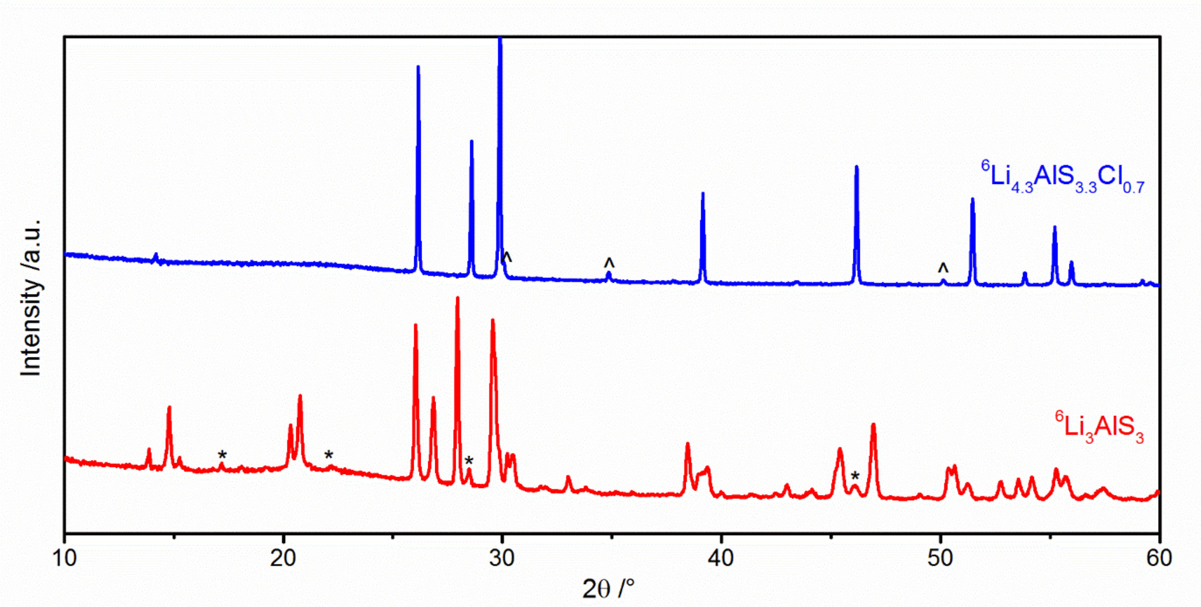

Figure S1. Laboratory XRD patterns of ^{6}Li enriched samples $^6Li_3AlS_3$ (red line) and $^6Li_{4.3}AlS_{3.3}Cl_{0.7}$ (blue line) obtained with λ = 1.5406 Å. The phases contain small amounts of impurities: Li_5AlS_4 for Li_3AlS_3 (*) and LiCl for $Li_{4.3}AlS_{3.3}Cl_{0.7}$ (^).

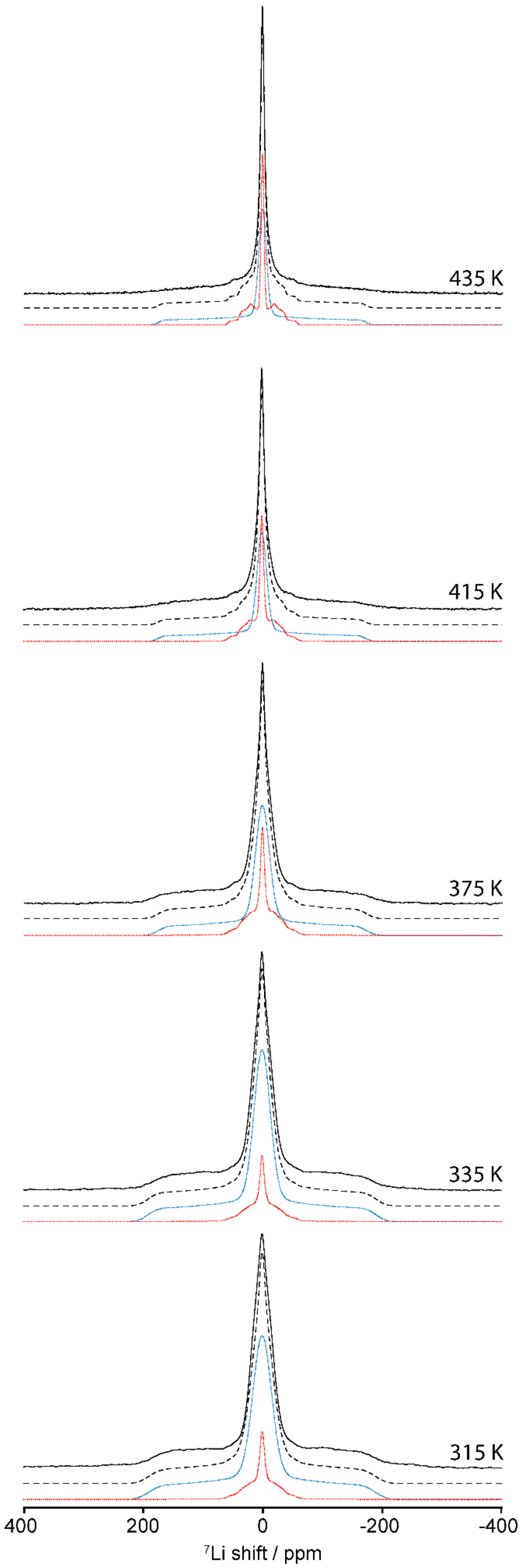

Figure S2. Static ^{7}Li one pulse NMR spectra of Li_3AlS_3 at selected temperatures demonstrating how the two components of the lineshape vary as a function of temperature. Experimental spectra (full black line), total fit (dashed black line) and spectral deconvolution for the broad (blue dotted line) and narrow (red dotted line) are shown.

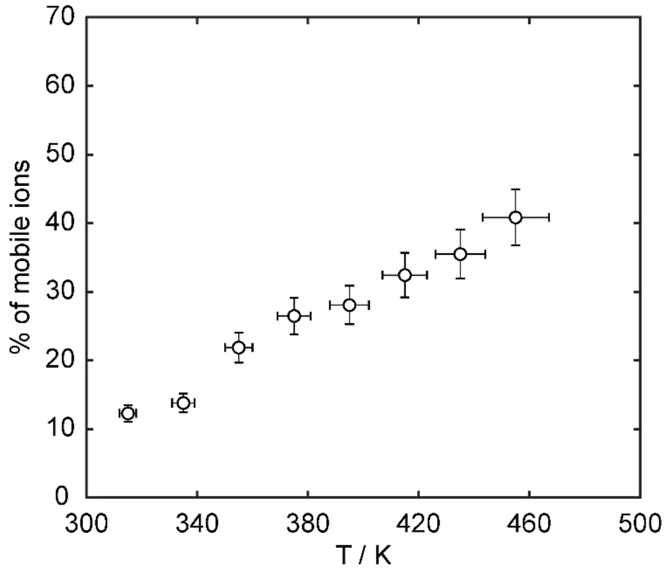

Figure S3. Percentage of mobile ions present in Li_3AlS_3 as a function of temperature. The percentage of mobile ions was taken as the contribution from the narrow component to the overall integration of the static ^{7}Li resonance.

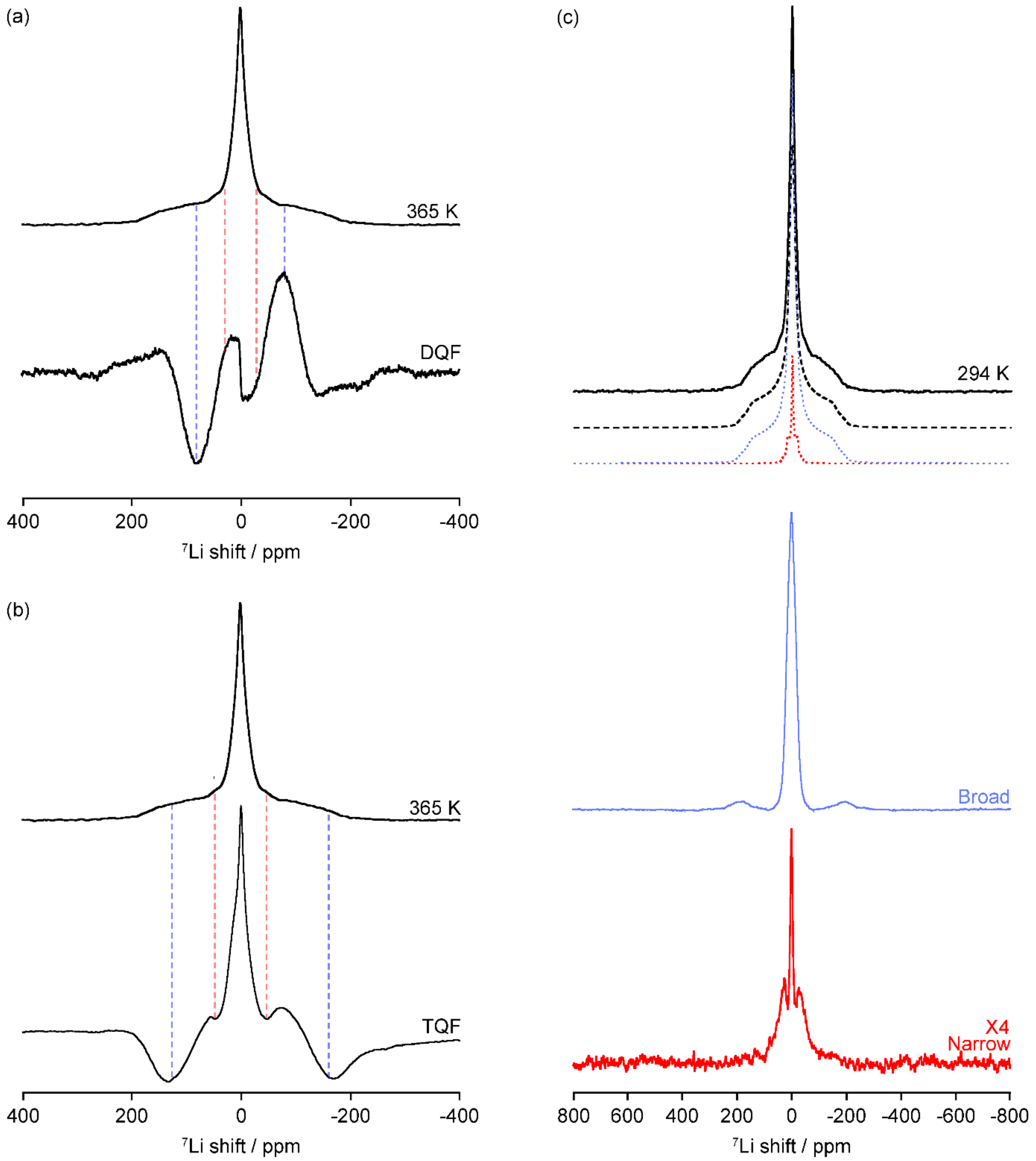

Figure S4. ^{7}Li NMR spectra of Li_3AlS_3 recorded at 9.4 T under static conditions. (a) Double-quantum filtered (DQF)[1–3] spectrum at 365 K, where the central transition is supressed and the quadrupolar satellite transitions associated with the ± 3/2 <-> ± 1/2 transitions have opposite phase. (b) Triple-quantum filtered (TQF) spectrum at 365 K in which the quadrupolar satellites have inverted phase. The coloured dashed lines highlight the satellite transitions associated with the broad component (blue) and narrow component (red), respectively. (c) Hahn-echo pulse sequence at room temperature with two different dephasing delays (9 and 90 μs in the blue and red spectra, respectively), allowing for the observation of the two separate components. The ^{7}Li one pulse spectra at the corresponding temperatures are also shown, where in panel (c) the experimental spectrum (full black lines), total fit (dashed black lines) and spectral deconvolution of the broad component (blue dotted line) and narrow component (red dotted line) are given.

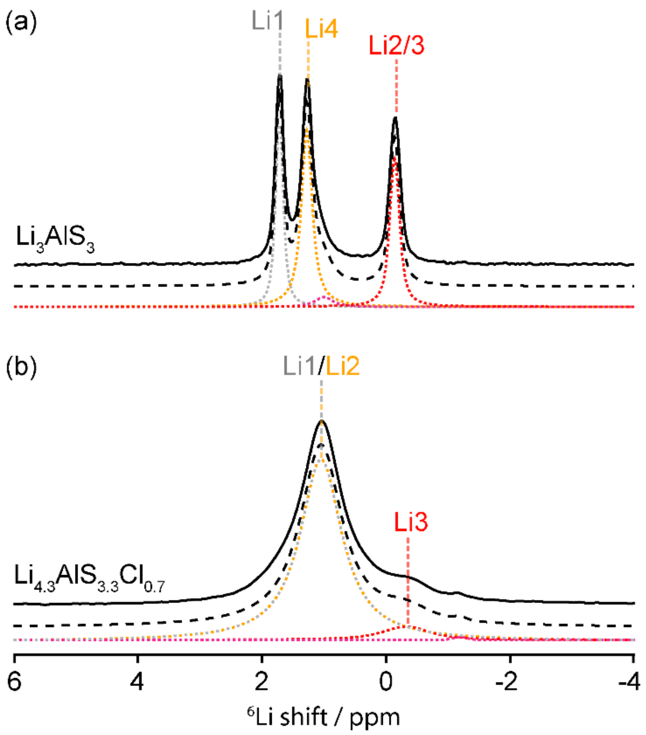

Figure S5. Room temperature ^{6}Li MAS one pulse NMR spectra collected at 20 T and $\omega_r/2\pi$ = 20 kHz of (a) Li_3AlS_3 and (b) $Li_{4.3}AlS_{3.3}Cl_{0.7}$. The experimental spectra (full lines), total fit (dashed lines), spectral deconvolution of each Li1/Li2/Li3/Li4 signals (dotted lines), impurities (Li_5AlS_4[4] in Li_3AlS_3 and solid LiCl[5] in $Li_{4.3}AlS_{3.3}Cl_{0.7}$ as per the powder X-ray-diffraction patterns in Figure S1, pink dotted lines) and spectral assignments are shown. Previously reported data for Li_3AlS_3[6] and $Li_{4.3}AlS_{3.3}Cl_{0.7}$[7] are provided here for comparison between both phases.

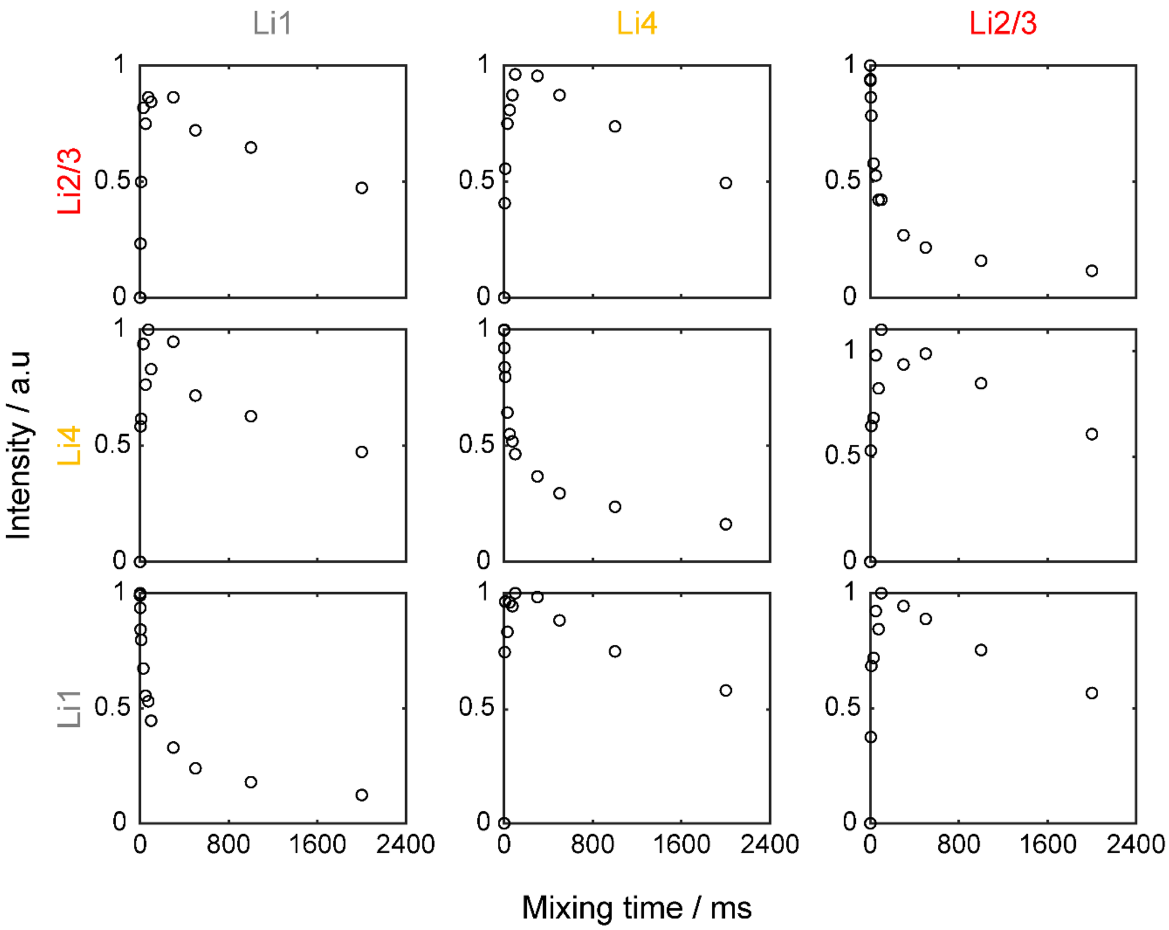

Figure S6. Intensity of the diagonal and cross-peaks in the ^{6}Li-^{6}Li EXSY NMR spectra of Li_3AlS_3 at $\omega_r/2\pi$ = 45 kHz as a function of the mixing time τ_m. The columns and rows are labelled on the left and the top according to the different resonances.

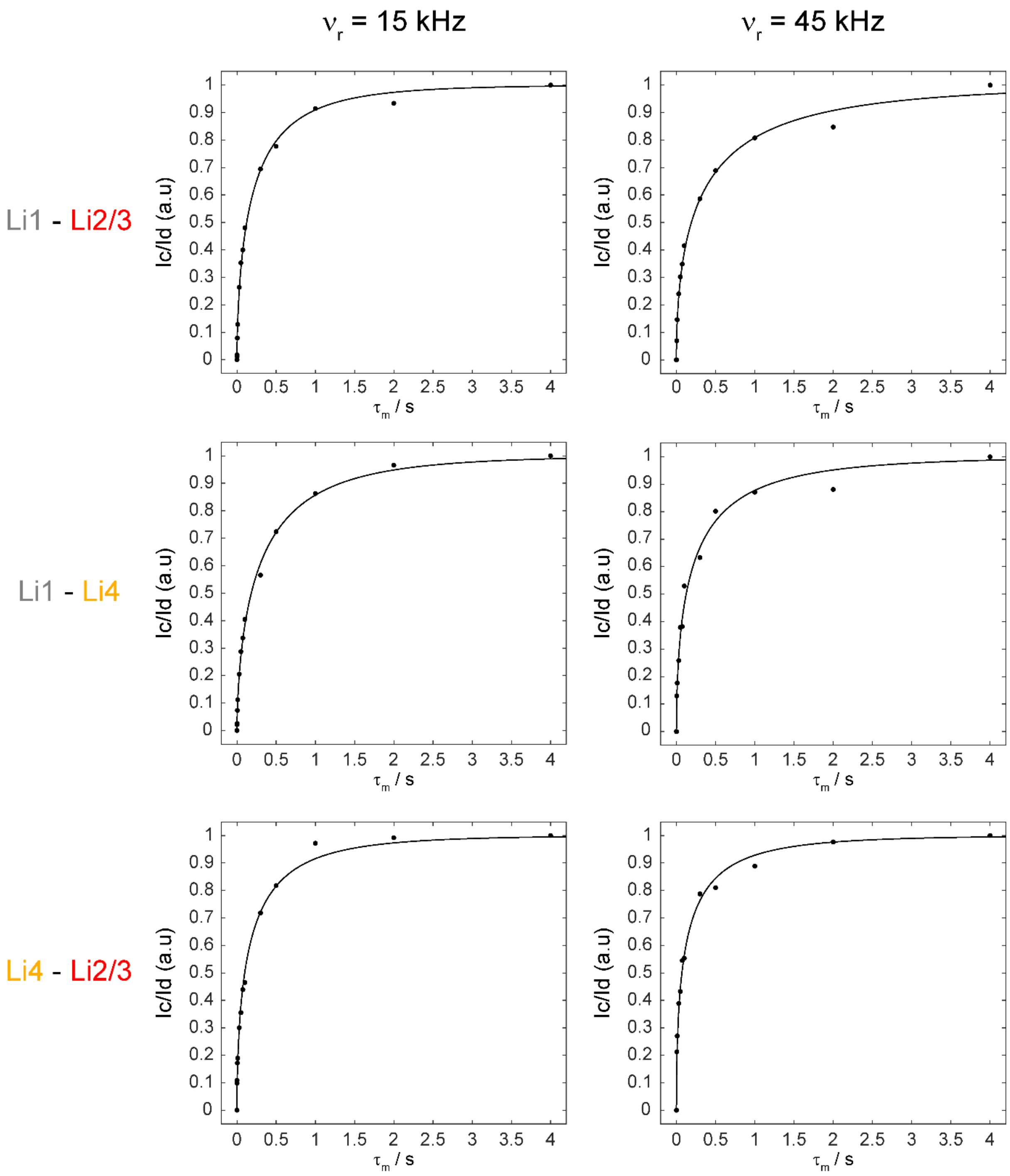

Figure S7. Plots of the ratio of the cross-peaks (Ic) *vs.* diagonal intensities (Id) in the ^{6}Li-^{6}Li EXSY NMR spectra of Li_3AlS_3 at MAS frequencies $\omega_r/2\pi$ = 15 (left) and 45 kHz (right) as a function of τ_m. The solid lines correspond to fits of the data according to equation 4.

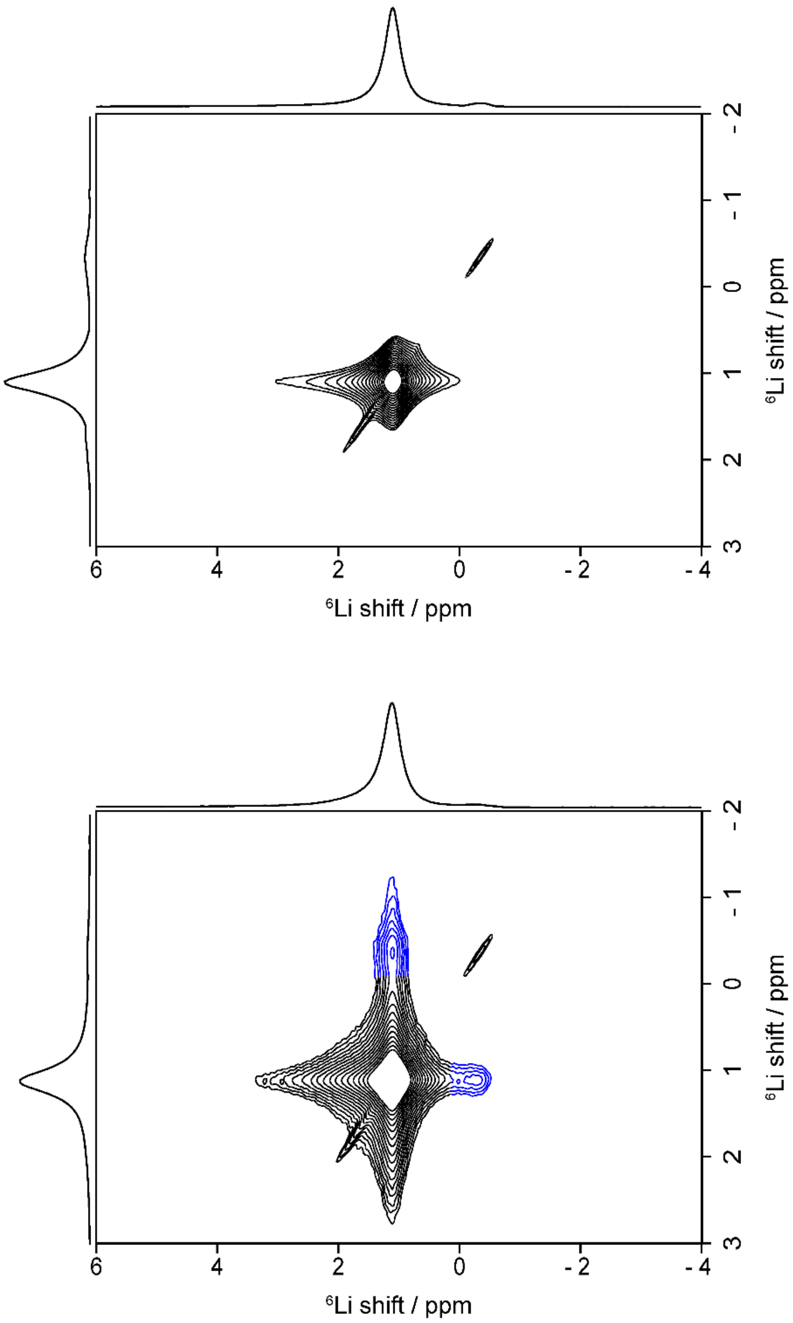

Figure S8. Examples of ^{6}Li-^{6}Li EXSY NMR spectra of ^{6}Li enriched $Li_{4.3}AlS_{3.3}Cl_{0.7}$ recorded at $\omega_r/2\pi$ = 45 kHz and τ_m of 0 (top) and 0.5 s (bottom) with diagonal and cross peaks shown in black and blue, respectively.

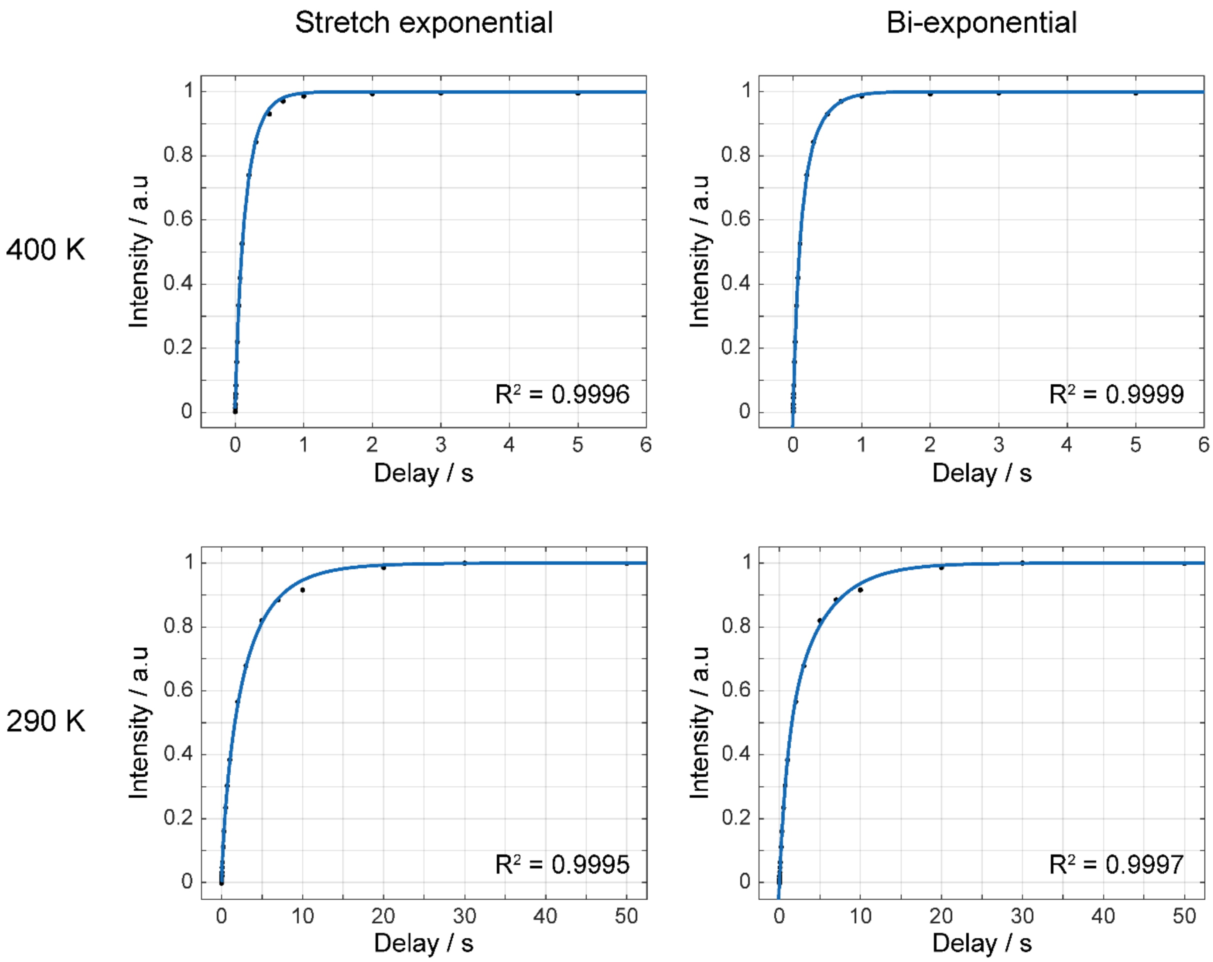

Figure S9. Comparison of methods to fit ${}^7Li$ T_1 build up curves of Li_3AlS_3 at representative temperatures of 290 and 400 K, where the data was fit with a stretch exponential of the form $1-\exp[-(\tau/T_1)^{\alpha}]$ (left) and a bi-exponential of the form $1-a\cdot\exp[-(\tau /T_{1,slow})]+b\cdot\exp[-(\tau/T_{1,fast})]$ (right). The goodness of fit (R^2 values) are given in the figure and illustrate slightly better fit in the case of a bi-exponential.

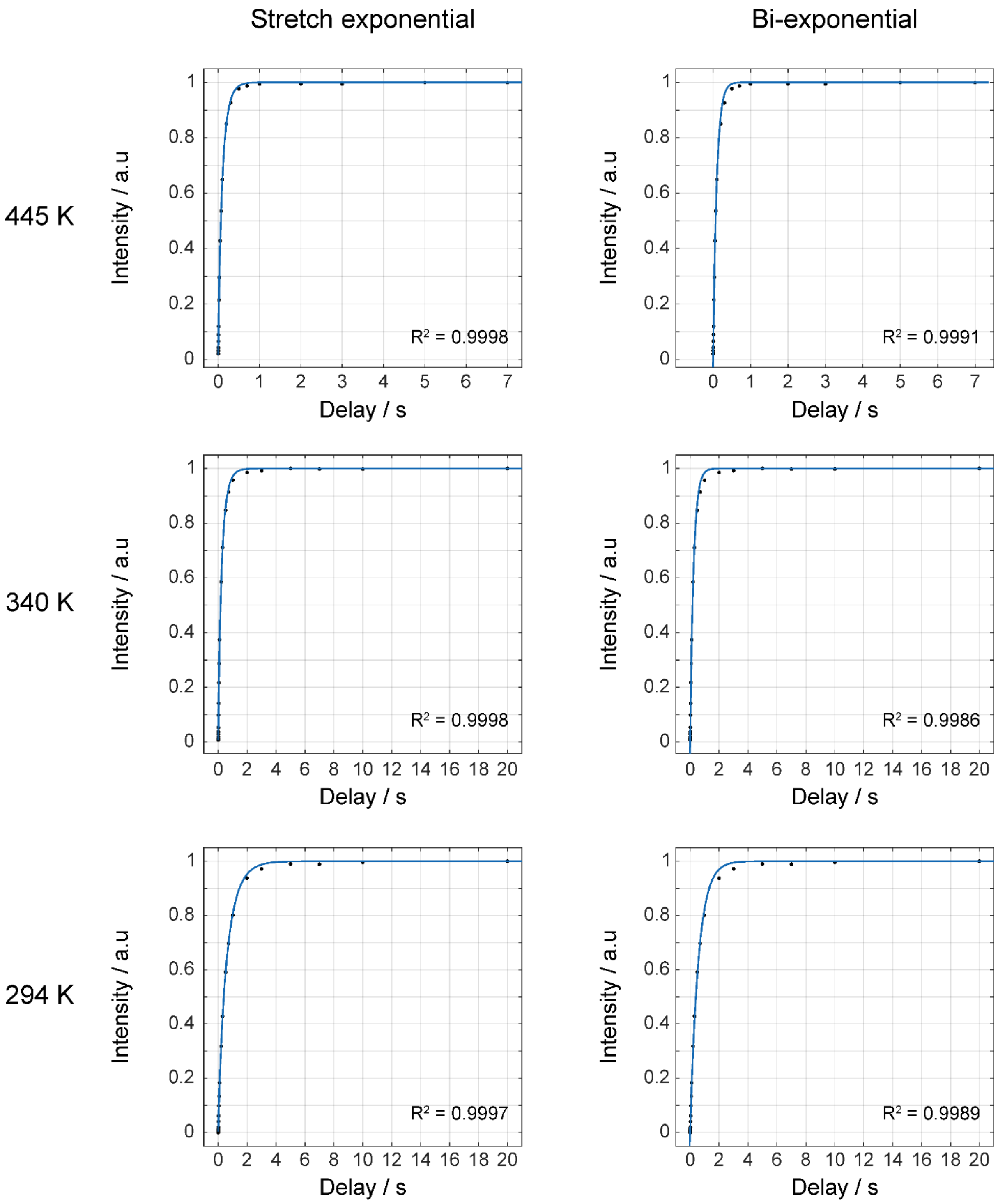

Figure S10. Comparison of methods to fit ^{7}Li T_1 build up curves of $Li_{4.3}AlS_{3.3}Cl_{0.7}$ at representative temperatures of 294, 340 and 445 K, where the data was fit with a stretch exponential of the form $1-\exp[-(\tau/T1)^{\alpha}]$ (left) and a bi-exponential of the form $1-a\cdot\exp[-(\tau/T_{1,slow})]+b\cdot\exp[-(\tau/T_{1,fast})]$ (right). The goodness of fit (R^2 values) are given in the figure and illustrate slightly better fit in the case of a stretch exponential.

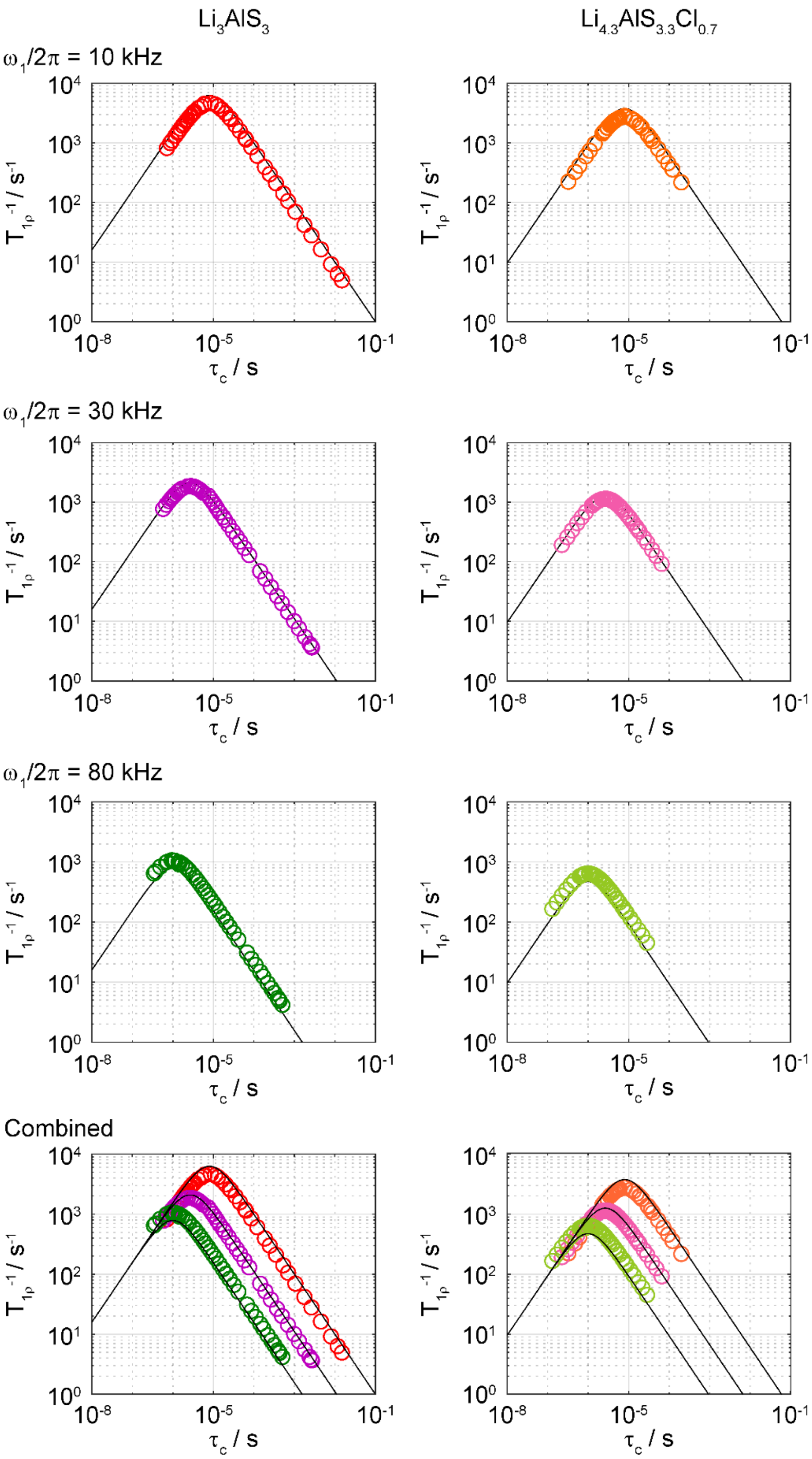

Figure S11. ^7Li $T_{1\rho}^{-1}$ versus τ_c for Li_3AlS_3 (left) and $Li_{4.3}AlS_{3.3}Cl_{0.7}$ (right). Data were collected at spin-lock frequencies of $\omega_1/2\pi$ of 10 kHz (red and orange), 30 kHz (purple and pink) and 80 kHz (green and olive) and the solid lines are those obtained from equation 10 using the experimentally determined local field fluctuation terms of 1.1(6) x 10^9 and 6.3(8) x 10^8 Hz^2 for Li_3AlS_3 and $Li_{4.3}AlS_{3.3}Cl_{0.7}$ respectively. Averaged values over the three $\omega_1/2\pi$ were used resulting in the slight offsets observed at high τ_c.

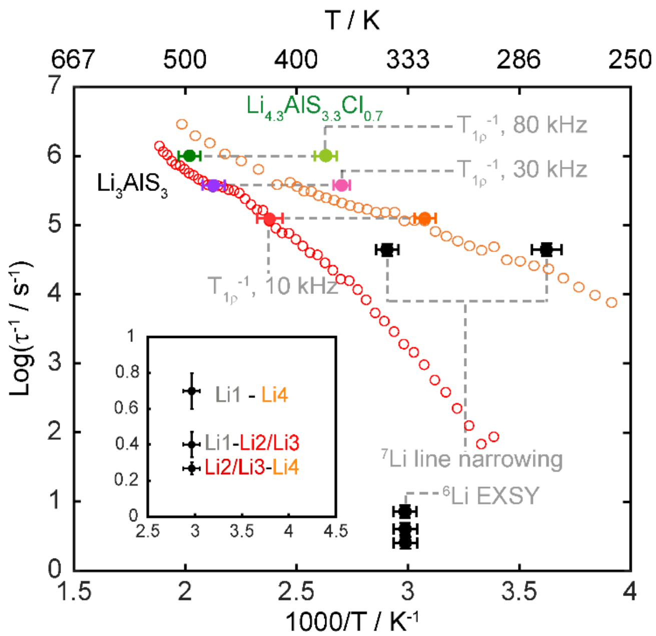

Figure S12. Arrhenius plot of Li ion jump rates extracted from ^{7}Li linewidth, ^{7}Li SLR in the rotating frame and 2D ^{6}Li-^{6}Li EXSY experiments and Bloembergen-Purcell-Pound (BPP) simulations red and orange open circles (10 kHz), for Li_3AlS_3 and $Li_{4.3}AlS_{3.3}Cl_{0.7}$, respectively.

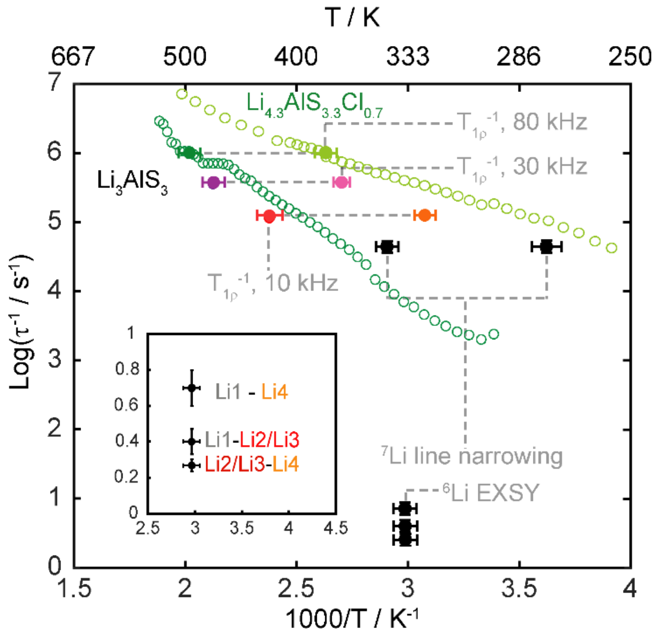

Figure S13. Arrhenius plot of Li ion jump rates extracted from ^{7}Li linewidth, ^{7}Li SLR in the rotating frame and 2D ^{6}Li-^{6}Li EXSY experiments and BPP simulations green and olive open circles (80 kHz), for Li_3AlS_3 and $Li_{4.3}AlS_{3.3}Cl_{0.7}$, respectively.

Table S1. Summary of the shortest interatomic distances between the various crystallographic Li sites in Li_3AlS_3 and $Li_{4.3}AlS_{3.3}Cl_{0.7}$ extracted from diffraction data.[6,7] Note the labelling of the Li sites varies between the two materials, with Li2 referring to octahedral Li in Li_3AlS_3, while Li2 corresponds to tetrahedral Li in $Li_{4.3}AlS_{3.3}Cl_{0.7}$.

Sample	Interatomic distance / Å					
	Li1-Li2	Li1-Li3	Li1-Li4	Li2-Li3	Li2-Li4	Li3-Li4
Li_3AlS_3	4.07(13)	3.12(3)	3.322(13)	3.3(3)	4.49(8)	2.83(3)
$Li_{4.3}AlS_{3.3}Cl_{0.7}$	1.274(14)	3.074(4)	N/A	2.406(5)	N/A	N/A

Table S2. Summary of the activation energies for Li_3AlS_3 and $Li_{4.3}AlS_{3.3}Cl_{0.7}$ extracted from AC Impedance spectroscopy (ACIS),[6,7] ^{7}Li motional narrowing[8] from Figure 3, spin-lattice relaxation data in the laboratory frame (T_1) and the rotating frame ($T_{1\rho}$) from Figure 7. Activation energies on both the high and low temperature flanks are quoted as well as the activation energy obtained from the jump rate plot (Figure 8).

Sample	Activation Energy /eV					
	ACIS	Waugh-Fedin	T_1	$T_{1\rho,LT}$	$T_{1\rho,HT}$	Jump rate
Li_3AlS_3	0.48(1)	~0.5	0.32(6)	0.42(8)	0.52(8)	0.29 (0.15-0.41)
$Li_{4.3}AlS_{3.3}Cl_{0.7}$	0.33(1)	~0.4	0.15(5)	0.19(4)	0.33(5)	0.29 (0.25-0.37)

References

(1) Jaccard, G.; Wimperis, S.; Bodenhausen, G. Multiple-Quantum NMR Spectroscopy of S=3/2 Spins in Isotropic Phase: A New Probe for Multiexponential Relaxation. *Interact. J. Chem. Phys.* **1986**, *85*, 4546.

(2) Eliav, U.; Navon, G. Measurement of Dipolar Interaction of Quadrupolar Nuclei in Solution Using Multiple-Quantum NMR Spectroscopy. *J. Magn. Reson. - Ser. A* **1996**, *123* (1), 32–48.

(3) Huynh, T. V.; Messinger, R. J.; Sarou-Kanian, V.; Fayon, F.; Bouchet, R.; Deschamps, M. Restricted Lithium Ion Dynamics in PEO-Based Block Copolymer Electrolytes Measured by High-Field Nuclear Magnetic Resonance Relaxation. *J. Chem. Phys.* **2017**, *147* (13), 134902.

(4) Lim, H.; Kim, S.-C.; Kim, J.; Kim, Y.-I.; Kim, S.-J. Structure of Li_5AlS_4 and Comparison with Other Lithium-Containing Metal Sulfides. *J. Solid State Chem.* **2018**, *257*, 19–25.

(5) Meyer, B. M.; Leifer, N.; Sakamoto, S.; Greenbaum, S. G.; Grey, C. P. High Field Multinuclear NMR Investigation of the SEI Layer in Lithium Rechargeable Batteries. *Electrochem. Solid-State Lett.* **2005**, *8* (3), 145–148.

(6) Gamon, J.; Duff, B. B.; Dyer, M. S.; Collins, C.; Daniels, L. M.; Surta, T. W.; Sharp, P. M.; Gaultois, M. W.; Blanc, F.; Claridge, J. B.; Rosseinsky, M. J. Computationally Guided Discovery of the Sulfide Li_3AlS_3 in the Li–Al–S Phase Field: Structure and Lithium Conductivity. *Chem. Mater.* **2019**, *31* (23), 9699–9714.

(7) Gamon, J.; Dyer, M. S.; Duff, B. B.; Vasylenko, A.; Daniels, L. M.; Zanella, M.; Gaultois, M. W.; Blanc, F.; Claridge, J. B.; Rosseinsky, M. J. $Li_{4.3}AlS_{3.3}Cl_{0.7}$: A Sulfide-Chloride Lithium Ion Conductor with Highly Disordered Structure and Increased Conductivity. *Chem. Mater.* **2021**, *33* (22), 8733–8744.

(8) Waugh, J. S.; Fedin, E. I. Determination of Hindered-Rotation Barriers in Solids. *Sov. Phsics-Solid State* **1963**, *4* (8), 1633–1636.
